# Whole-blood point-of-care glial fibrillary acidic protein and ubiquitin C-terminal hydrolase L1 biomarkers after traumatic brain injury in the US: a prospective diagnostic prediction of intracranial injury on head CT

**DOI:** 10.1016/j.lana.2026.101539

**Published:** 2026-07-20

**Authors:** Ava M. Puccio, Michael A. McCrea, David O. Okonkwo, Swati Pradhan-Bhatt, Kenneth Curley, Adam Moss, Manish Gupta, Erica Wyse, Deeksha Pahwa, Damien Hoffman, Claudia Robertson, Joseph Giacino, Ramon Diaz-Arrastia, Nancy Temkin, Esther Yuh, Pratik Mukherjee, Raj Chandran, Geoffrey T. Manley, Krista Caudle, Beth McQuiston

**Affiliations:** a Department of Neurological Surgery, University of Pittsburgh, Pittsburgh, PA, USA; b Department of Neurosurgery, Medical College of Wisconsin, Milwaukee, WI, USA; c Abbott Laboratories, Abbott Park, IL, USA; d Iatrikos Research and Development Strategies, Allied Technologies and Consulting (ATC) Supporting Defense Health Agency, Operational Medical Systems (OPMED), Fort Detrick, MD, USA; e Defense Health Agency, Operational Medical Systems (OPMED), Fort Detrick, MD, USA; f Department of Neurosurgery, Baylor College of Medicine, Houston, TX, USA; g Department of Physical Medicine and Rehabilitation, Harvard Medical School, Boston, MA, USA; h Department of Neurology, University of Pennsylvania, Perelman School of Medicine, Philadelphia, PA, USA; i Departments of Neurological Surgery and Biostatistics, University of Washington, Seattle, WA, USA; j Department of Neurological Surgery, University of California San Francisco, San Francisco, CA, USA

**Keywords:** TBI, Glasgow Coma Scale, TBI blood test, Biomarkers, Guidelines, CT risks, Imaging, Intracranial lesions, GFAP, UCH-L1, Neurological outcome, Health economics and outcomes of TBI, Acute presentation of TBI, TBI guidelines, TBI consensus, Concussion guidelines, TBI policy, Diagnostic TBI testing, Point-of-care TBI testing

## Abstract

**Background:**

Biomarkers like glial fibrillary acidic protein (GFAP) and ubiquitin carboxy-terminal hydrolase L1 (UCH-L1) are being globally recommended as a valuable component in the evaluation of acute TBI. Having a regulatory-approved whole-blood test is imperative to provide consistent and democratized approach to healthcare. This prospective, national multicenter cohort trial (NCT04171960) evaluated the performance of a whole-blood, point-of-care (POC) GFAP/UCH-L1 panel in determining need for head computed tomography (CT) in adult patients presenting to the emergency department (ED) with suspected traumatic brain injury (TBI).

**Methods:**

Adults presenting to the ED with suspected TBI, Glasgow Coma Scale score 13–15, completed head CT scan, and blood collected within 24 h of injury were included. GFAP and UCH-L1 measured in whole-blood through a POC TBI biomarker test were analyzed using prespecified cutoffs and combined into a single test result and compared with CT findings.

**Findings:**

970 enrolled participants [mean age 46.8 (18–97); 36%female; 69%White, 18%Black; 19%Hispanic/Latino] were analyzed. 683 participants (70.4%) had ‘elevated’ and 287 (29.6%) had ‘not elevated’ TBI blood test result. For detection of CT intracranial injury, the whole-blood TBI test had sensitivity of 96.5% (95%CI 93.6–98.1%) and NPV 96.5% (95%CI 93.7–98.1%).

**Interpretation:**

The GFAP/UCH-L1 whole-blood test had high sensitivity and NPV for prediction of intracranial injury within 24 h of TBI. The platform received U.S.-FDA clearance and expands clinical use of the TBI test beyond the laboratory. This test provides results in 15 min; supporting rapid triage and decision-making in suspected TBI.

**Funding:**

Defense Health Agency (DHA) under Contract No. W81XWH19C0071.

**Research in context**

**Evidence before this study**

Large multicenter plasma studies, including the pivotal ALERT TBI trial and subsequent TRACK TBI and CENTER TBI analyses, demonstrated that GFAP and UCH-L1 provide high sensitivity and high negative predictive value for identifying CT positive intracranial injury in adults with suspected mild TBI. These findings supported the first FDA cleared serum and plasma assays and established strong clinical and analytical validity for biomarker guided CT decision making in emergency care. Serum and plasma testing offered robust and reproducible performance in controlled laboratory settings, enabling regulatory clearance and inclusion in national and international guidelines. Building on this foundation, whole blood point of care testing has emerged to extend biomarker use into rapid, decentralized, and resource constrained clinical environments where immediate decision making is essential. Databases searched for this manuscript included: PubMed, Medline, ClinicalTrials.gov and Scopus from 2018 to present with the following keywords: TBI; Glasgow Coma Scale; TBI blood test; biomarkers; guidelines; CT risks; imaging; intracranial lesions; GFAP; UCH-L1; neurological outcome; health economics and outcomes of TBI; acute presentation of TBI; TBI guidelines; TBI consensus; concussion guidelines; TBI policy; diagnostic TBI testing; point-of-care TBI testing.

**Added value of this study**

This large prospective multicenter regulatory trial evaluated a whole blood point-of-care GFAP and UCH-L1 test using a prespecified cutoff algorithm in adults with suspected mild TBI across 20 trauma centers. The test demonstrated high sensitivity and high negative predictive value for detecting CT positive intracranial injury within 24 h of injury and correctly identified all neurosurgical level lesions. Results were available in approximately 15 min without the need for centrifugation.

Implications of all the available evidence: These findings supported FDA clearance of the whole blood point of care platform and provide new evidence for its feasibility and operational utility in emergency and resource limited settings.

**Implications of all the available evidence**

This point-of-care blood test allows rapid measurement of brain injury markers which may in the future assist clinicians in the evaluation of TBI. The use of biomarkers has already been incorporated into global guidelines for TBI assessment and will continue to be expanded as healthcare systems adopt more objective approaches to brain injury care. Integration of biomarkers within the recent, updated NIH-NINDS guidelines, specifically the CBI-M framework, further strengthens its role by contributing data alongside clinical, imaging and modifier domains. Bringing accurate testing closer to patients empowers clinicians with objective data and helps democratize access to high-quality TBI evaluation across diverse clinical settings, assisting in resource utilization and elevating healthcare access for all. The blood test potentially supports future health by decreasing radiation due to unnecessary CT scans and resultant increased concomitant cancer risk. Next steps include establishing pediatric-specific guidelines and biomarker-related use cases to direct triage, treatment and prevention across the lifespan.

## Introduction

Recent scientific breakthroughs and international consensus initiatives are transforming clinical management of traumatic brain injury (TBI). This has included the emergence of blood-based biomarkers to aid in the evaluation and characterization of acute TBI.^1^, ^2^, ^3^ The National Institutes of Health (NIH) and National Institute of Neurological Disorders and Stroke (NINDS) have spearheaded the development of a multidimensional framework, known as the Clinical, Biomarker, Imaging, and Modifier (CBI-M) model,^4^, ^5^, ^6^, ^7^, ^8^, ^9^, ^10^, ^11^ which integrates multiple domains to more precisely characterize TBI across mild, moderate, and severe presentations. In alignment with other national and international guidelines and consensus statements,^12^, ^13^, ^14^, ^15^, ^16^, ^17^, ^18^, ^19^, ^20^ this progression positions biomarkers such as glial fibrillary acidic protein (GFAP) and ubiquitin carboxy-terminal hydrolase L1 (UCH-L1) as a valuable component in the evaluation of acute TBI. These biomarkers have demonstrated high accuracy for detection of intracranial injury visible on computed tomography (CT) scanning in multiple large-scale studies, including the ALERT-TBI^21^^,^^22^ study, and multi-site research efforts: TRACK-TBI (Transforming Research and Clinical Knowledge in Traumatic Brain Injury),^23^, ^24^, ^25^, ^26^ and CENTER-TBI (Collaborative European NeuroTrauma Effectiveness Research in TBI).^27^

Complementary publications from the NIH NINDS Blood-Based Biomarkers Working Group^8^ and the European TBI Consensus Paper^12^ reinforce the clinical utility of GFAP and UCH-L1 for triage and clinical evaluation, and advocate for their integration into emergency department workflows. Studies show that biomarker-guided CT reduction strategies are not only clinically effective but also cost-efficient, with favorable pricing thresholds demonstrated in U.S. models and per-patient savings estimated in France.^28^ While CT imaging remains a cornerstone of acute evaluation, it also contributes substantially to cumulative radiation exposure. Projected estimates show that current CT utilization in the U.S. could result in over 100,000 future cancers, with CT-related malignancies potentially comprising up to 5% of all new cancer diagnoses.^29^^,^^30^

GFAP and UCH-L1 are now FDA-cleared for use on both core laboratory and POC platforms (Abbott Laboratories, FDA K201778, K234143)^21^^,^^22^^,^^31^, ^32^, ^33^, ^34^ and are formally recognized in the 2024 American College of Surgeons Trauma Quality Program (ACS TQP) Best Practices Guidelines^13^ for reducing unnecessary CT imaging in patients with suspected TBI and in international guidelines.^12^^,^^17^^,^^19^^,^^35^ Sample preparation for these assays, however, requires blood centrifugation, which requires processing in core laboratory facilities, increasing the test turnaround time and limiting clinical implementation.

Improving ease and speed of sample acquisition and processing is a key advance for clinical adoption of TBI biomarker testing. Using whole blood and extending the diagnostic window to 24 h post-injury would enable testing in a wider range of acute assessment settings (e.g., military medicine, urgent care, prehospital environments), while eliminating the need to centrifuge a blood sample after venipuncture. A whole blood i-STAT POC TBI blood test could also be performed at the bedside directly. With the potential for use in additional diverse settings, this innovation supports faster and more accessible TBI evaluation while reducing unnecessary CT scans and radiation exposure.

This prospective, multicenter cohort regulatory trial evaluated the clinical performance of a whole-blood, POC GFAP/UCH-L1 biomarker panel to assist in determining the need for a head CT scan in adult patients (GCS score 13–15) presenting with suspected TBI.

## Methods

### Study design and setting

This national study was conducted through the Transforming Research and Clinical Knowledge in Traumatic Brain Injury (TRACK-TBI) network, which included enrolment at 20 clinical sites at Level 1 and 2 trauma centers across the United States (Supplementary Table S1) from July 2020 through June 2023. Approval was obtained from an accredited Institutional Review Board (IRB) for each of the participating sites and the study was conducted in accordance with the principles of the Declaration of Helsinki and all applicable regulatory requirements (U.S. regulations 21 CFR parts 50, 54, 56 and 812). This study was registered at ClinicalTrials.gov (Identifier: NCT04171960) prior to patient enrollment.

### Subject enrollment

Participants were screened in the ED for having a plausible mechanism of injury for acute TBI and having a head CT performed as part of clinical care. TBI was operationally defined and adjudicated based on the original American Congress of Rehabilitation Medicine (ACRM) criteria.^36^ Participants were required to exhibit at least one of the following manifestations of trauma-induced brain disruption: a period of loss of consciousness, retrograde or antegrade amnesia, altered mental state at time of injury, or a transient or current focal neurological deficit. Screening of participants occurred by research personnel using the inclusion criteria of: 1) age ≥ 18 years; 2) presenting to the emergency department (ED) with suspected TBI within 12 h of injury (to ensure that a blood draw would be obtained within 24 h of injury); 3) initial GCS score of 13–15; and 4) head CT performed as part of their standard clinical care. Participants were not eligible for the study if: 1) they were diagnosed with an ischemic or hemorrhagic stroke; 2) time of injury was not known; 3) presented with penetrating head trauma or spinal cord injury (with American Spinal Injury Association [ASIA] score of C or worse); 4) standard of care head CT scan would not be completed prior to ED or hospital discharge; or 5) were prisoners or patients in custody or patients on psychiatric hold. Participants were screened for competency and the ability to provide written, informed consent through speech intelligibility and Galveston Orientation and Amnesia Test (GOAT) assessments. Depending on the outcome of competency assessment, and if they met all the inclusion criteria and none of the exclusion criteria, the participant or their legally authorized representative (LAR) provided written consent to participate in the study. At any point in the study, the participant, or LAR, could withdraw their consent for participation. Demographic data, including race and ethnicity, were self-reported by participants.

### Neuroimaging

For all study participants, a non-contrast head CT scan was performed per standard-of-care. The CT images were de-identified and randomly assigned using randomization tables generated by a statistician to two American Board of Radiology certified neuroradiologists to conduct an independent, blinded review of each CT scan without access to any other clinical or laboratory data of the participant, except for age and gender. The NIH Common Data Elements (CDE)^37^ were reported for each CT scan, and it was classified as ‘positive’, ‘negative’ or ‘inconclusive’ for presence of acute intracranial lesions consistent with TBI, using the Marshall classification system.^38^ Presence of at least one of the following acute findings, indicated an intracranial lesion: cortical contusion, intracerebral hematoma, epidural hematoma, subarachnoid hemorrhage, acute and acute-on-chronic subdural hematoma, intraventricular hemorrhage, traumatic vascular injury, traumatic axonal injury, diffuse axonal injury, diffuse cerebral swelling, and brain herniation. CT findings noting a Marshall CT Classification category VI, i.e., presence of a high or mixed density lesion >25 mL (not evacuated), were categorized as a neurosurgical lesion (potentially requiring neurosurgical intervention). If both initial reviewers provided concordant classifications of the CT scan, the adjudicated result was considered final. If consensus was not achieved, the CT scan was independently reviewed by a third board-certified neuroradiologist, who was blinded to the initial assessments, to establish consensus classification. If there was no agreement among the three reviewers, the CT scan was adjudicated as ‘inconclusive’.

### Blood biomarker measurements

A venous whole blood specimen was prospectively collected from each enrolled study participant within 24 h of injury. Specimens were tested by trained personnel using the i-STAT TBI cartridges and i-STAT Alinity Instruments (with investigational software: FRT2239_TBI_WB_CS1 with TBI CLEW; expiration date: 31Dec2025). Specimen testing was initiated within 60 min of specimen collection time. The i-STAT TBI cartridge is a multiplex immunoassay that contains assays for simultaneous measurement of both GFAP and UCH-L1. The assays test for the presence of these biomarkers in a single whole blood sample and yield a semi-quantitative test interpretation based on the measurements of GFAP and UCH-L1 in approximately 15 min. The reportable range for GFAP in whole blood is 47 pg/mL to 10,000 pg/mL, and that for UCH-L1 is 87 pg/mL to 3200 pg/mL. Operators performing i-STAT testing were blinded to the independent CT evaluation results as assessed by the study neuroradiologists, and they were not informed of the GFAP and UCH-L1 assay cut-offs. The facilities used at each clinical site and the research study staff who performed the testing were representative of POC end users.

### Blood biomarker analysis and testing

For the tested specimens, measured GFAP and UCH-L1 concentrations were used to report a semi-quantitative test interpretation (‘Elevated’/‘Not Elevated’/no valid test interpretation) based on whether the GFAP and the UCH-L1 results were at, above or below their respective cut-offs for a ‘positive’ intracranial lesion on a CT scan (65 pg/mL for GFAP; 360 pg/mL for UCH-L1) as shown in Table 1. The cut-offs were determined prior to the study and designed to maximize sensitivity and negative predictive value (NPV).

**Table 1:** i-STAT TBI test interpretation matrix from individual assay results relative to cut-offs.

GFAP assay result (relative to cutoff of 65 pg/mL)	UCH-L1 assay result (relative to cutoff of 360 pg/mL)	TBI test interpretation
Equal or Above	Equal or Above	Elevated
Equal or Above	Below	Elevated
Equal or Above	∗∗∗ or Not Reported	Elevated
Below	Equal or Above	Elevated
Below	Below	Not Elevated
Below	∗∗∗ or Not Reported	No valid test interpretation
∗∗∗ or Not Reported	Equal or Above	Elevated
∗∗∗ or Not Reported	Below	No valid test interpretation
∗∗∗ or Not Reported	∗∗∗ or Not Reported	No valid test interpretation

The test was repeated if no valid test interpretation was reported on the instrument.

Clinical performance of the i-STAT TBI test was assessed by comparing the i-STAT TBI test interpretation with the participant-matched head CT scan result. Sensitivity, specificity, NPV, positive predictive value (PPV), positive likelihood ratio (pos. LR) and negative likelihood ratio (neg. LR) of the i-STAT TBI test, with their respective confidence intervals (CIs) were calculated.

### Statistical analyses

In conjunction with the regulatory body requirements, sample size was estimated using the Wilson Score test based on the allowable width of the 95% confidence interval for a proportion. Assuming a 96.5%-point estimate for sensitivity with the lower bound of the Wilson score 95% confidence interval of no less than 90%, the sample size was estimated to be a minimum of 110 head CT positive samples. Assuming a 34%-point estimate for specificity with the lower bound of the Wilson score 95% confidence interval no less than 30%, the sample size was estimated to be a minimum of 570 head CT negative samples. Statistical analyses were performed using validated statistical software (Analyse-it, version 5.90; Minitab, version 21; and R, version 3.5.3).

### Role of funders

Abbott Point of Care (Abbott) served as the study sponsor and provided financial support. DHA and Abbott contributed to study design and provided technical and regulatory input. Study monitoring was performed by Abbott in accordance with regulatory requirements. Data analysis and interpretation were conducted by the authors, as detailed in the Authors’ contributions section. Authors from Abbott and the DHA contributed as co-authors in accordance with standard authorship criteria. DHA was provided the opportunity to review the manuscript for comment to the principal investigator, but not for approval or disapproval. All authors had full access to the data, take responsibility for the integrity of the data and the accuracy of the analysis, and retained final responsibility for the decision to submit for publication. No author received payment for writing this manuscript.

## Results

### Study participant characteristics

**Fig. 1:**
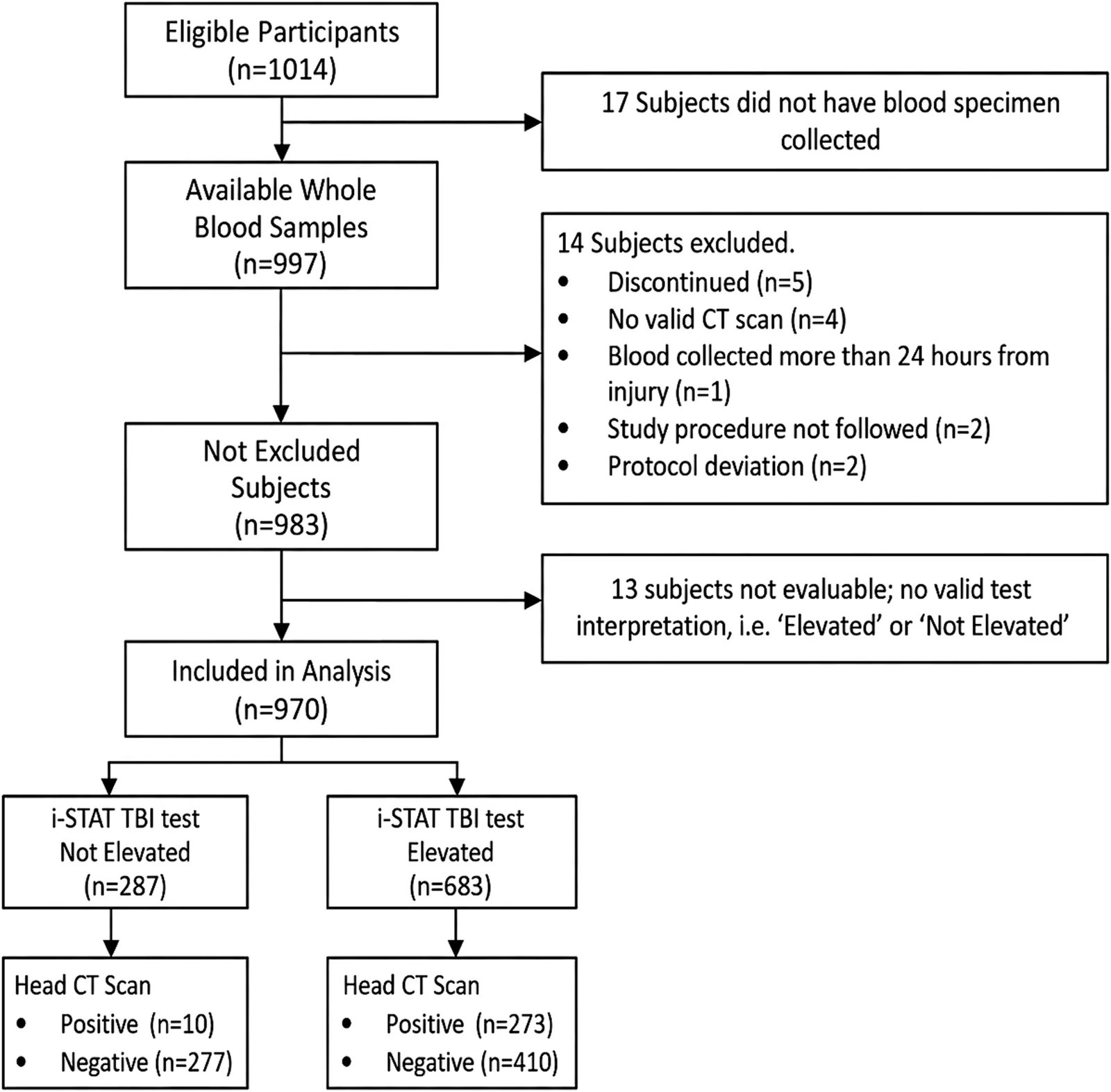
**Consolidated standards of reporting trials (CONSORT) diagram summarizing the study flow.** CT (computed tomography); TBI (traumatic brain injury). A total of 1207 subjects were screened for eligibility in this study. Of these, 101 subjects did not meet the eligibility criteria and were screen failed. Reasons included: • No informed consent collected due to a deviation from the study enrollment workflow including not collecting an ICF from the subject or LAR within a 5-day timeframe (99 subjects). • All inclusion criteria not met, or exclusion criteria identified prior to enrollment (2 subjects).

A total of 1014 participants were eligible and enrolled in the study, with whole blood specimens available for analysis in 997 (Fig. 1). Of these, 5 participants were discontinued from the study because either the participant withdrew their consent for participation or they were found to be ineligible post-enrollment based on the study inclusion/exclusion criteria. An additional 9 participants were excluded from the study because of a technically invalid CT scan or read as ‘inconclusive’ scans, blood collection after 24 h from injury, or the study protocol not being followed during specimen collection/testing. Specimens tested from 13 participants yielded invalid i-STAT TBI test interpretations. All data from the above participants were excluded from analysis. The remaining 970 participants had a valid i-STAT TBI test interpretation (Elevated’ or ‘Not Elevated’) with a valid adjudicated head CT scan result (‘Positive’ or ‘Negative’) and their data was included in the analysis represented in the current study.

The evaluable participants were predominantly Caucasian (68.6%) and male (64%) with a mean age of 46.8 ± 19.33 years of age (Table 2). The majority of participants were injured as a result of a direct impact to the head, a fall, or acceleration/deceleration type of injury, with 69.6% reporting a loss of consciousness (LOC). In 636 (65.6%) participants, physical examination revealed signs of visible trauma above the clavicle; and 44 (4.5%) presented with signs of basal skull fracture. Of the 970 participants, 762 (78.6%) had a GCS score of 15 and 208 (21.4%) had a GCS score of 13–14 (Table 2). There were no sex- or ethnicity-based statistical differences between the groups analyzed. There were no deaths reported in participants that had a negative TBI blood test result within the 6-month follow-up study timeframe.

**Table 2:** Demographic and baseline characteristics of participants.

Characteristics	Participants with GCS 13–15 (N = 970)	Participants with GCS 15 (n = 762)	Participants with GCS 13–14 (n = 208)
Age, mean yrs (SD) [range]	46.8 (19.33) [18, 97]	46.5 (19.30) [18, 97]	47.8 (19.47) [18, 95]
Male, No. (%)	621 (64.0)	480 (63.0)	141 (67.8)
Female, No. (%)	349 (36.0)	282 (37.0)	67 (32.2)
Race/Ethnicity, No. (%)			
White	665 (68.6)	528 (69.3)	137 (65.9)
Black or African American	172 (17.7)	139 (18.2)	33 (15.9)
Asian	49 (5.1)	34 (4.5)	15 (7.2)
Native Hawaiian/Pacific Islander	10 (1.0)	7 (0.9)	3 (1.4)
American Indian or Alaska Native	12 (1.2)	10 (1.3)	2 (1.0)
Other (Mixed Race)	13 (1.3)	8 (1.0)	5 (2.4)
Not Reported/Unknown	49 (5.1)	36 (4.7)	13 (6.3)
Hispanic or Latino	188 (19.4)	141 (18.5)	47 (22.6)
GCS score, No. (%)			
13	39 (4.0)	0 (0.0)	39 (18.8)
14	169 (17.4)	0 (0.0)	169 (81.2)
15	762 (78.6)	762 (100.0)	0 (0.0)
Mechanism of injury, No. (%)			
Acceleration/Deceleration	289 (29.8)	235 (30.8)	54 (26.0)
Direct impact (blow to head)	129 (13.3)	93 (12.2)	36 (17.3)
Direct impact (head against object)	594 (61.2)	467 (61.3)	127 (61.1)
Fall (ground level)	252 (26.0)	200 (26.2)	52 (25.0)
Fall (from height > 1 m)	118 (12.2)	93 (12.2)	25 (12.0)
Crush	3 (0.3)	3 (0.4)	0 (0.0)
Blast	0 (0.1)	1 (0.1)	0 (0.0)
Other	22 (2.3)	17 (2.2)	5 (2.4)
Neurological assessment, No. (%)			
Loss of Consciousness (LOC)	675 (69.6)	518 (68.0)	157 (75.5)
Confusion/Alteration of consciousness (AOC)	699 (72.1)	525 (68.9)	174 (83.7)
Vomiting	45 (4.6)	31 (4.1)	14 (6.7)
Post traumatic amnesia (PTA)	605 (62.4)	440 (57.7)	165 (79.3)
Post traumatic seizures	3 (0.3)	0 (0.0)	3 (1.4)
Physical evidence, No. (%)			
Visible trauma above clavicle	636 (65.6)	477 (62.6)	159 (76.4)
Signs of basal skull fracture	44 (4.5)	25 (3.3)	19 (9.1)
Intoxicated with drugs, No. (%)			
Yes	114 (11.8)	75 (9.8)	39 (18.8)
Intoxicated with alcohol, No. (%)			
Yes	110 (11.3)	62 (8.1)	48 (23.1)

Blood was drawn at a median of 8.1 h after injury (Table 3). For 283 participants (29.2%), the head CT showed the presence of one or more acute intracranial lesion(s). Of these, 14 participants (1.4%) had a neurosurgical lesion (potentially requiring neurosurgical intervention). Common head CT findings were subarachnoid hemorrhages (77%), acute subdural hematomas (62.5%) and contusions (41.3%).

**Table 3:** TBI biomarker blood test results.

Test results	Participants with GCS 13–15 (N = 970)	Participants with GCS 15 (n = 762)	Participants with GCS 13–14 (n = 208)
Hours from injury to blood draw, median, [range], (IQR)	8.1 [1.5, 24.0] (4.4, 14.9)	6.6 [1.5, 24.0] (4.1, 13.7)	11.8 [2.2, 24.0] (6.8, 17.3)
GFAP, median, [range], (IQR) pg/ml			
CT positive	739.0 [10.0, 10000.0] (341.0, 1716.0)	568.5 [10.0, 10000.0] (275.8, 1284.5)	1172.0 [30.0, 10000.0] (569.5, 3062.0)
CT negative	78.0 [0.0, 5240.0] (27.0, 236.0)	73.0 [0.0, 5240.0] (25.0, 212.0)	136.0 [0.0, 3437.0] (43.5, 524.0)
UCH-L1, median [range], (IQR) pg/ml			
CT positive	211.0 [13.0, 2672.0] (131.0, 395.0)	192.0 [28.0, 2006.0] (128.5, 308.0)	283.0 [13.0, 2672.0] (150.3, 525.8)
CT negative	164.0 [15.0, 3200.0] (99.0, 279.0)	160.0 [16.0, 2558.0] (98.2, 264.0)	196.0 [15.0, 3200.0] (112.0, 371.0)
Positive test, No. (%)	283 (29.2)	176 (23.1)	107 (51.4)
UCH-L1, blood draw within 3 h from injury, n, median [range], (IQR) pg/ml	**N = 70**	**N = 58**	**N = 12**
CT positive (blood draw within 3 h from injury)	598.5 [133.0, 1976.0] (253.1, 1041.0)	674.0 [133.0, 956.0] (223.2, 909.0)	523.0 [264.0, 1976.0] (307.2, 1733.8)
CT negative (blood draw within 3 h from injury)	279.5 [34.0, 2558.0] (131.8, 469.9)	280.0 [34.0, 2558.0] (223.2, 909.0)	236.0 [83.0, 568.0] (149.7, 409.3)

### Clinical performance results

**Fig. 2:**
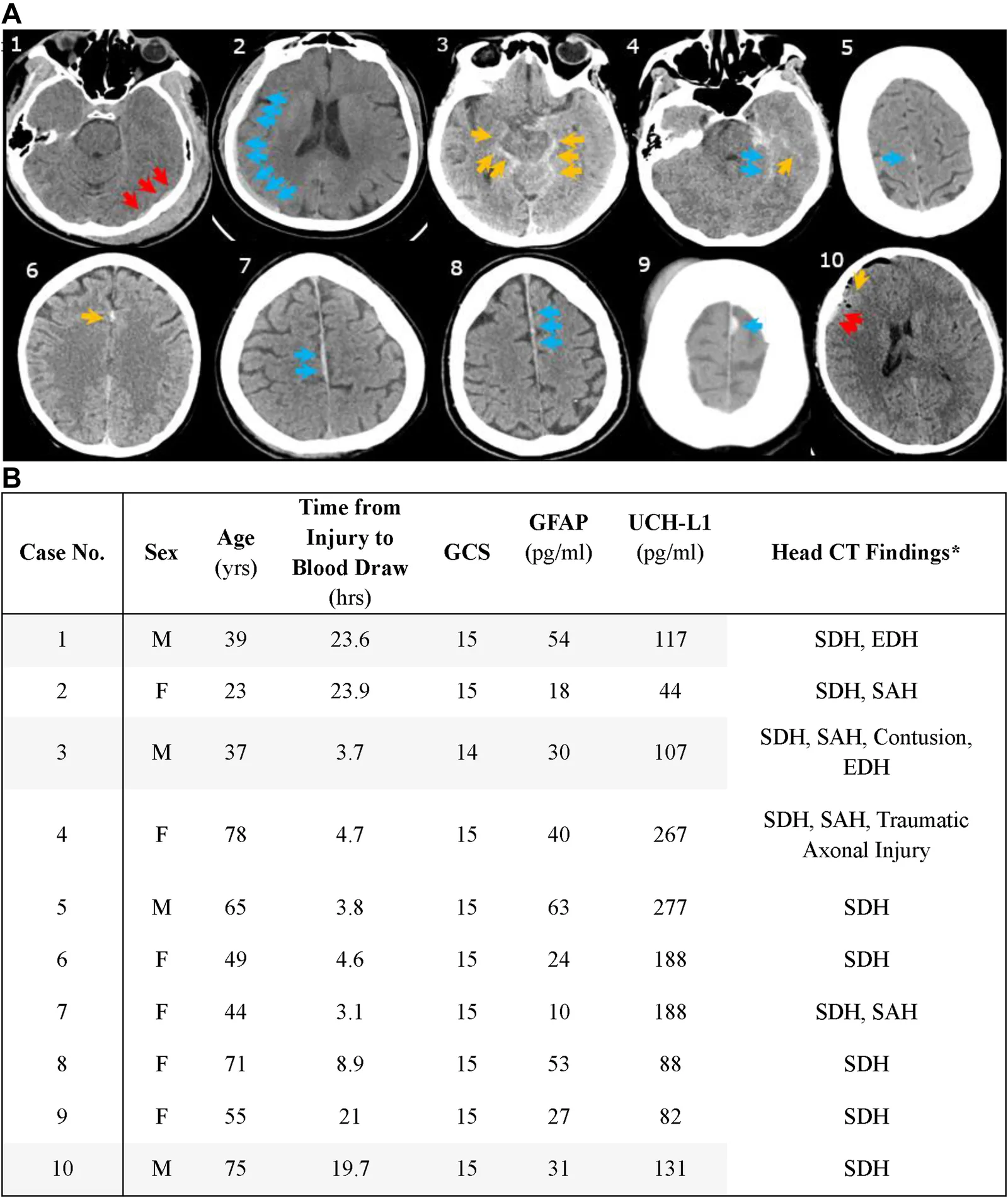
**Head CT images and characteristics of participants with false negative test results (n = 10).** A: Non-contrast head CT images of ten participants with false negative results. Red arrows indicate epidural hematoma; blue arrows indicate subdural hematoma; orange arrows indicate subarachnoid hemorrhage. B:∗Subdural hematoma (SDH), subarachnoid hemorrhage (SAH), epidural hematoma (EDH). No acute intracranial lesion was >25 mL (i.e., did not require neurosurgical intervention) and all head CT scans showed normal cisterns.

Of the 970 evaluable participants with a GCS score of 13–15, 683 (70.4%) had an ‘Elevated’ and 287 (29.6%) had a ‘Not Elevated’ i-STAT TBI test result. Among those with an ‘Elevated’ result, 273 (40%) had a positive CT scan (Supplementary Table S2). Ten participants had a false negative (FN) biomarker test result (i.e., blood test was below GFAP and UCH-L1 thresholds, but head CT demonstrated positive intracranial findings). Thus, the i-STAT TBI test had a sensitivity of 96.5% (95% CI: 93.6, 98.1) (Table 4). The rate of false negative (FN) results was 3.5%. None of the 10 participants with false negative test results required a surgical intervention related to their head injury as none of the identified lesions met the generally accepted standard of care surgical criteria.^39^ A single DICOM image best reflective of the CT abnormality of each of these participants is shown in Fig. 2.

**Table 4:** Diagnostic performance of the i-STAT point-of-care TBI Test for head CT findings in mild TBI patients.

Performance Characteristic	Participants with GCS 13–15 (n = 970)	Participants with GCS 15 (n = 762)	Participants with GCS 13–14 (n = 208)
Sensitivity, % (95% CI)	96.5% (93.6, 98.1)	94.9% (90.6, 97.3)	99.1% (94.9, 99.8)
Specificity, % (95% CI)	40.3% (36.7, 44.0)	42.5% (38.6, 46.5)	27.7% (19.9, 37.1)
Positive Predictive Value, % (95% CI)	40.0% (38.4, 41.5)	33.1% (31.4, 34.9)	59.2% (56.2, 62.1)
Negative Predictive Value, % (95% CI)	96.5% (93.7, 98.1)	96.5% (93.6, 98.1)	96.6% (79.5, 99.5)
Positive Likelihood Ratio (95% CI)	1.62 (1.52, 1.73)	1.65 (1.53, 1.79)	1.37 (1.23, 1.58)
Negative Likelihood Ratio (95% CI)	0.09 (0.05, 0.16)	0.12 (0.06, 0.22)	0.03 (0.01, 0.19)

Of the 287 participants associated with a ‘Not Elevated’ i-STAT TBI test result, 277 (96.5%) had a negative CT scan. Among the 683 participants with an ‘Elevated’ result, 410 (60%) had a negative CT scan (Supplementary Table S2). Thus, the i-STAT TBI test had a specificity of 40.3% (95% CI: 36.7,44.0) and a negative predictive value (NPV) of 96.5% (95% CI: 93.7,98.1). Participants with a GFAP or UCH-L1 result above the respective 90th percentiles, as found in this study (1581 pg/mL, 630 pg/mL, respectively) were 13 times more likely to have CT findings compared to participants below the GFAP and UCH-L1 cutoffs of 65 pg/mL and 360 pg/mL, respectively. Fourteen participants were identified with a lesion which may potentially require neurosurgical intervention and all 14 were correctly classified by the i-STAT TBI test with an ‘Elevated’ test interpretation (true positive).

## Discussion

In this prospective, multicenter cohort regulatory trial, a rapid whole-blood, POC TBI biomarker test combining GFAP and UCH-L1 had highly favorable performance of rapid blood-based biomarker testing for prediction of the absence of acute traumatic intracranial injury on head CT (rule-out head CT) up to 24 h following trauma in participants with a GCS score of 13–15. In patients presenting to the hospital ED with what is commonly classified as “mild TBI” based on a GCS score of 13–15, the i-STAT test had a sensitivity of 96.5% in identifying those with a positive head CT and negative predictive value of 96.5% in predicting the absence of traumatic intracranial injury on head CT within 24 h of injury. Importantly, the i-STAT test results were elevated above clinical cutoffs in 100% of cases with brain lesions potentially requiring emergent neurosurgical intervention.

The combination of high sensitivity and negative predictive value observed in the current study are critical to supporting the adoption of new diagnostic testing technologies that minimize the risk of false negative results, particularly in this context of use for predicting the absence of intracranial injury and guiding clinical decisions about the need for head CT in patients with suspected TBI. Moreover, these findings provide further support for the clinical utility of blood-based biomarkers in TBI patients with a GCS score of 13–15, who not only represent more than 90% of the TBI population and account for hundreds of thousands of ED visits every year in the US,^40^^,^^41^ but also often present the greatest diagnostic challenge to emergency medicine clinicians due to the relative subjectivity and subtlety of injury.

There remains a critical need for objective blood-based biomarkers to aid in diagnosing TBI at the milder end of the TBI continuum. Findings from the present study demonstrate substantial potential to reduce unnecessary head CT scans in patients with GCS 13–15. While a positive CT head remains an important clinical indicator and the initial diagnostic tool guiding TBI care in most settings, it is well recognized that some patients have intracranial hemorrhages detectable on MRI but not visible on CT.^42^ As such, a sizable subset of CT-negative patients may harbor structural abnormalities below the detection threshold of standard emergency department CT imaging.

Based on its demonstrated performance, the i-STAT point-of-care platform received U.S. Food and Drug Administration (FDA) regulatory clearance in 2024. Findings from the current study further promote the clinical utility of the TBI blood test for deployment in the acute and critical care settings, beyond the traditional laboratory setting, such as ED and urgent care clinic environments. Implementation in community hospitals and concussion clinics with CLIA-certification could substantially broaden access to blood-based biomarker testing across ED, military, and sports injury populations affected by TBI. The use of whole blood enables a streamlined workflow—from venipuncture to clinical result within 15 min, eliminating the need for laboratory centrifugation and expediting clinical decision-making.

In the combat casualty care settings, the operational utility and logistical burden of medical capabilities are key determinants for managing service members with TBI across echelons of care; especially when the combat casualty care pathway requires spanning distances of up to 100 km from Role 1 battlefield medic or battalion aid station, back to Role 3 field hospital for head CT. The chaos of the battlefield including high numbers of casualties necessitate straightforward objective methods for informing triage decisions that can sort casualties into three categories: don’t need immediate attention, need attention, and need immediate attention.^43^^,^^44^ Currently, over-triage of mild TBI in deployed settings is observed with high rates of aeromedical transport to Role 3 for head CT, and these unnecessary evacuations negatively impact the strength of the fighting force.^45^ The POC TBI test qualitative output has utility in forward military roles, in mass-casualty and other resource-constrained environments where obtaining a head CT after injury could be complicated by long-distance evacuation in hazardous conditions and/or limited availability of CT scanners.

The i-STAT Alinity POC device, when used with TBI assay cartridges, enables local medical providers to triage GCS 13–15 casualties objectively and determine the need for head CT or appropriate management and evacuation.^46^ This allows more appropriate prioritization of limited evacuation and imaging resources, potentially saving lives of more severely injured service members with polytrauma. The use of the TBI blood test fielded to operational deployed forces is distinct from non-deployed or civilian care; it’s a matter of warfighting strategy as future near-peer large scale combat operations will preclude air superiority and traditional evacuation chains will simply be unavailable.^47^ The POC TBI blood test may keep service members further forward, allowing for faster return to duty and combat readiness.

This trial builds on a robust body of evidence demonstrating the utility of GFAP and UCH-L1 in the management of TBI. These biomarkers have consistently correlated with intracranial abnormalities detected on CT and with injury severity across the TBI continuum. The ALERT-TBI trial, was pivotal in establishing their clinical relevance and led to the FDA’s 2018 clearance of a serum-based assay combining GFAP and UCH-L1 to predict the absence of intracranial injury on head CT. Subsequent investigations have confirmed that biomarker combination achieves sensitivities ranging from 95.8% to 100% and NPVs from 99.3% to 100% for ruling out traumatic intracranial injury in patients with GCS scores of 13–15.^48^

The current GFAP/UCH-L1 thresholds were established to optimize negative predictive value for head CT in patients with GCS 13–15 and clinical evidence of TBI—parameters most applicable to US health care settings. At these thresholds, corresponding positive predictive value is relatively low. In ALERT-TBI, fewer than 5% of positive CT head scans among patients with GCS 14–15 revealed neurosurgical manageable findings,^21^ and the necessity of intensive health care resource use for minor traumatic injuries remains debated.^49^ In resource-limited settings, applying higher thresholds to define a positive test may offer greater clinical and operational utility.

In 2025, the NIH/NINDS Blood-Based Biomarkers Working Group further affirmed the clinical value of GFAP and UCH-L1, recommending their inclusion as core components of the proposed CBI-M Framework for characterization and classification of acute TBI.^4^^,^^8^ These biomarkers have been shown to perform similarly to established clinical decision rules, including the Canadian CT Head Rule and NEXUS II in predicting CT detectable abnormalities; achieving combined AUCs exceeding 0.80 in emergency department cohorts.^50^ Beyond diagnostic accuracy, GFAP and UCH-L1 also demonstrate prognostic significance, with elevated levels correlating with increased risk of neurosurgical intervention, mortality, and unfavorable outcomes at 6- and 12-months post-injury.^51^ The integration of GFAP and UCH-L1 in French and Scandinavian TBI guidelines,^20^^,^^52^ along with recent FDA clearance of POC platforms such as the i-STAT system, highlights their expanding role in real-world clinical practice. Collectively, these advances laid the foundation for regulatory validation and clinical implementation, culminating in the current trial’s demonstration of their feasibility for rapid, whole-blood testing at the POC and subsequent FDA clearance.

Several limitations are inherent to the data acquisition methods used in this regulatory trial. The study was conducted primarily at Level 1 trauma centers which admit higher acuity patients within a short interval from injury to ED presentation and are either direct admits or transfers due to concern for head CT abnormalities. The median time for blood testing was 8 h from injury, and participants were captured as early as possible by the research teams. With research enrolling constraints, 70 participants (7.2%) were able to be enrolled within an ultra-early timeframe (3 h of injury), which still allowed for representation of this earlier timeframe. Although this study was conducted in high acuity environments of level 1 and 2 trauma centers, the findings are promising and may be generalizable to a broader range of care settings. The ease of implementation of a laboratory test with whole blood eliminates the need for additional centrifugation (i.e., workflow, staff, equipment) in a hospital laboratory; however, technicians acquiring the blood samples should be trained to avoid hemolysis during venipuncture as this could influence UCH-L1 concentrations.^53^ This streamlined approach can expedite the future implementation of testing to all levels of acute and critical care, including austere environments allowing for more accurate triaging in military and remote contexts while reducing unnecessary interhospital transfers and associated costs through more objective assessment of potential CT abnormalities. Although sex and/or gender can influence TBI outcomes, a sex- and gender-based analysis was not conducted in this study. Future studies with larger, more diverse cohorts are warranted to explore potential sex- and gender-related differences in biomarker performance and clinical outcomes.

Beyond diagnostic applications, the availability of a validated point-of-care blood test for TBI marks a pivotal advancement in the broader field of neurotrauma research and care. Biomarkers such as GFAP and UCH-L1 not only facilitate rapid triage and imaging decisions but also provide objective measures for patient stratification in therapeutic and prevention trials. Their incorporation into clinical workflows paves the way for precision medicine approaches, including targeted pharmacologic interventions, neuroprotective strategies, and optimized nutritional support. Ongoing Department of Defense-sponsored drug trials exemplify how biomarker-guided enrollment, monitoring, and outcome assessment can enhance trial efficiency and interpretability. More broadly, the capacity to objectively evaluate brain injury in real time may accelerate the development of novel therapies and improve long-term outcomes across diverse populations, including athletes, military personnel, and civilians.

### Conclusion

This multicenter regulatory trial demonstrates that whole-blood, POC TBI biomarker testing combining GFAP and UCH-L1 on the i-STAT platform delivers high diagnostic accuracy, based on its sensitivity and negative predictive value for detecting traumatic intracranial injury within 24 h of trauma in patients with GCS scores of 13–15. With its rapid turnaround time, operational simplicity, and FDA clearance in 2024, the test represents a potentially transformative tool in emergency and acute care settings. Its demonstrated utility in patients with GCS 13–15, who comprise the majority of TBI cases and present substantial diagnostic challenges in the hospital ED, underscores its clinical relevance and potential to reduce unnecessary CT imaging, streamline triage, and improve patient outcomes. These results align with emerging consensus recommendations supporting the broader adoption of TBI biomarker testing across community hospitals, TBI clinics, and military environments, while also establishing a foundation for biomarker-guided therapeutic clinical trials in TBI. Expanding access to biomarker-based testing, especially with the emergence of a whole blood test that doubles the diagnostic window to 24 h and enables deployment in diverse care settings, offers a transformative opportunity to reduce radiation exposure across a broader patient population. By extending access to rural, prehospital, and military environments, this convergence of regulatory clearance, clinical validation, economic feasibility, and radiation safety signals a new era in TBI care—one defined by precision, efficiency, accessibility, and long-term health stewardship. As blood-based biomarkers become integrated into SOC, they are poised to fundamentally reshape the diagnosis, classification, and management of TBI, ushering in a new era of objective, accessible, and personalized neurotrauma care.

## Contributors

Conceptualization: KC (Curley), BM; Study design: AMP, DOO, JTG, RDA, KC (Caudle), BM, GTM, EY, MM, CR; Methodology: KC (Caudle), RC; Software: RC; Visualization: AMP, BM, EY; Data collection: AMP, DOO, JTG, RC, GTM, EY, CR, NT, PM; Data analysis: AMP, DOO, BM, RC, SPB, PM; Data interpretation: AMP, DOO, DH, MM, SPB, NT; Literature search: AMP, KC (Curley); Writing- original draft: AMP, DOO, BM, DP, RC, MM; Writing-review and editing: AMP, DOO, JTG, RDA, DH, KC (Curley), KC (Caudle), BM, DP, EW, MG, AM, GTM, MM, CR, NT; Project Administration: DH, KC (Curley), KC (Caudle), EW; Funding acquisition: KC (Caudle), BM; Project Supervision: MG, AM, BM; Resources: MG, AM; Site selection: GTM.

All authors reviewed and approved the final manuscript, had access to the data reported in the study and had final responsibility for the decision to submit for publication.

## Data sharing statement

Raw data requests can be made to the corresponding author, in conjunction with Abbott Laboratories and the United States Department of Defense.

## Declaration of interests

AMP reports grants and contracts from Abbott Laboratories. She has no other relevant conflicts of interest to declare. MAM reports research funding from Abbott Laboratories. DOO reports grants and contracts from Abbott Laboratories. SPB has no relevant conflicts of interest. KC (Curley) has no other relevant conflicts of interest to declare. AM, MG, EW, DP are employed by Abbott Laboratories. DH has no relevant conflicts of interest to declare. CR has no other relevant conflicts of interest to declare. JC has no other relevant conflicts of interest to declare. RDA reports grants and contracts from Abbott Laboratories. He has received equipment/materials/services from MesoScale Discoveries and has served as a consultant for Dompe Pharma and Stream Biomedical. NT has no relevant conflicts of interest to declare. EY has received grants/contracts from Abbott Laboratories (past 36 months). She has no other relevant conflicts of interest to declare. PM no relevant conflicts of interest to declare. RC is employed by Abbott. He holds Abbott stock and is lead inventor on numerous patents (assigned to Abbott; no personal royalties). GTM reports grants and contracts from Abbott Laboratories. He reports no support for the present manuscript from any commercial entity. KC (Caudle) has no relevant conflicts of interest to declare. BM is employed by Abbott Laboratories. She holds Abbott stock and is lead inventor on over 70 patents granted or pending (assigned to Abbott; no personal royalties).
