# Supplementary material for: Whole-blood point-of-care glial fibrillary acidic protein and ubiquitin C-terminal hydrolase L1 biomarkers after traumatic brain injury in the US: a prospective diagnostic prediction of intracranial injury on head CT
Source: Lancet Reg Health Am. 2026 Jul 20;61:101539. doi: 10.1016/j.lana.2026.101539 (PMC13575800; doi:10.1016/j.lana.2026.101539)
Supplement: Supplementary file 1 [file mmc1.pdf]

Whole-Blood Point-of-Care Glial Fibrillary Acidic Protein (GFAP) and Ubiquitin C-terminal Hydrolase L1 (UCH-L1) Biomarkers in Prediction of Intracranial Injury on Head CT after Traumatic Brain Injury

Supplementary Tables

Supplementary Table 1. Study Sites in the United States.

Supplementary Table 2. Performance of the i-STAT point-of-care TBI Test for Head CT scan findings in mild TBI patients (n=970) with GCS scores 13-15.

Supplementary Figures

Supplementary Figure 1. Boxplot of GFAP Values by CT Status.

Supplementary Figure 2. Boxplot of UCH-L1 Values by CT Status.

Appendix 1: Instructions for Use, the i-STAT TBI test

Supplementary Table 1: Study Sites in the United States

Site ID	Site Name
001	University of California, San Francisco<br>Brain and Spinal Injury Center<br>San Francisco, California
002	Baylor College of Medicine<br>Department of Neurosurgery<br>Houston, Texas
003	University of Pittsburgh Medical Center Presbyterian Hospital<br>Pittsburgh, Pennsylvania
004	University of Pennsylvania, Presbyterian Medical Center<br>Philadelphia, Pennsylvania
005	The University of Texas at Austin<br>Austin, Texas
006	University of Rochester Medical Center<br>Rochester, New York
007	University of Washington<br>Seattle, Washington
008	Medical College of Wisconsin<br>Milwaukee, Wisconsin
009	University of Maryland Medical Center<br>Baltimore, Maryland
010	University of Miami<br>Miami, Florida
011	University of Texas Southwestern Medical Center<br>Dallas, Texas
012	University of Utah<br>Salt Lake City, Utah
013	Massachusetts General Hospital<br>Boston, Massachusetts
014	Virginia Commonwealth University Health<br>Department of Emergency Medicine<br>Richmond, Virginia
015	Indiana University Methodist Hospital/ IU Health Neuroscience Center<br>Indianapolis, Indiana
016	Goodman Campbell Brain and Spine / St. Vincent Hospital<br>Carmel, Indiana
017	University of Kentucky<br>Department of Emergency Medicine<br>Lexington, Kentucky
018	University of Cincinnati Medical Center

Site ID	Site Name
	Cincinnati, Ohio
019	Denver Health and Hospital Authority<br>Denver, Colorado
021	University of Texas Health Sciences Center of Houston<br>Houston, Texas

*Sites were Level 1 or Level 2 Trauma centers.

Supplementary Table 2: Performance of the i-STAT point-of-care TBI Test for Head CT scan findings in mild TBI patients (n=970) with GCS scores 13-15

	Head CT Positive (N=283)	Head CT Negative (N=687)
i-STAT TBI Test 'Elevated' (N=683)	273	410
i-STAT TBI Test 'Not Elevated' (N=287)	10	277

Supplementary Figure 1. Boxplot of GFAP values by CT Status

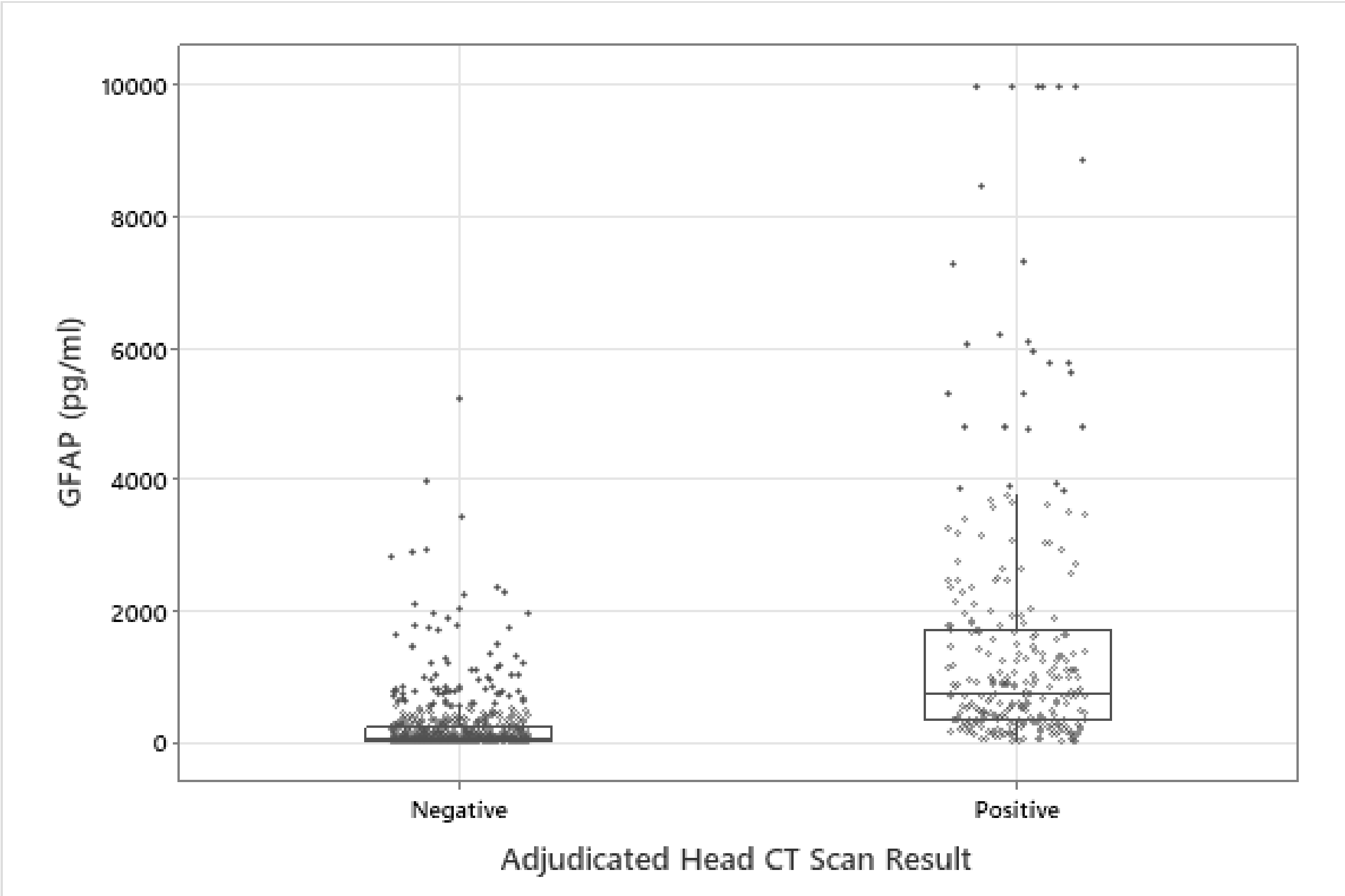

Supplementary Figure 2. Boxplot of UCH-L1 values by CT Status

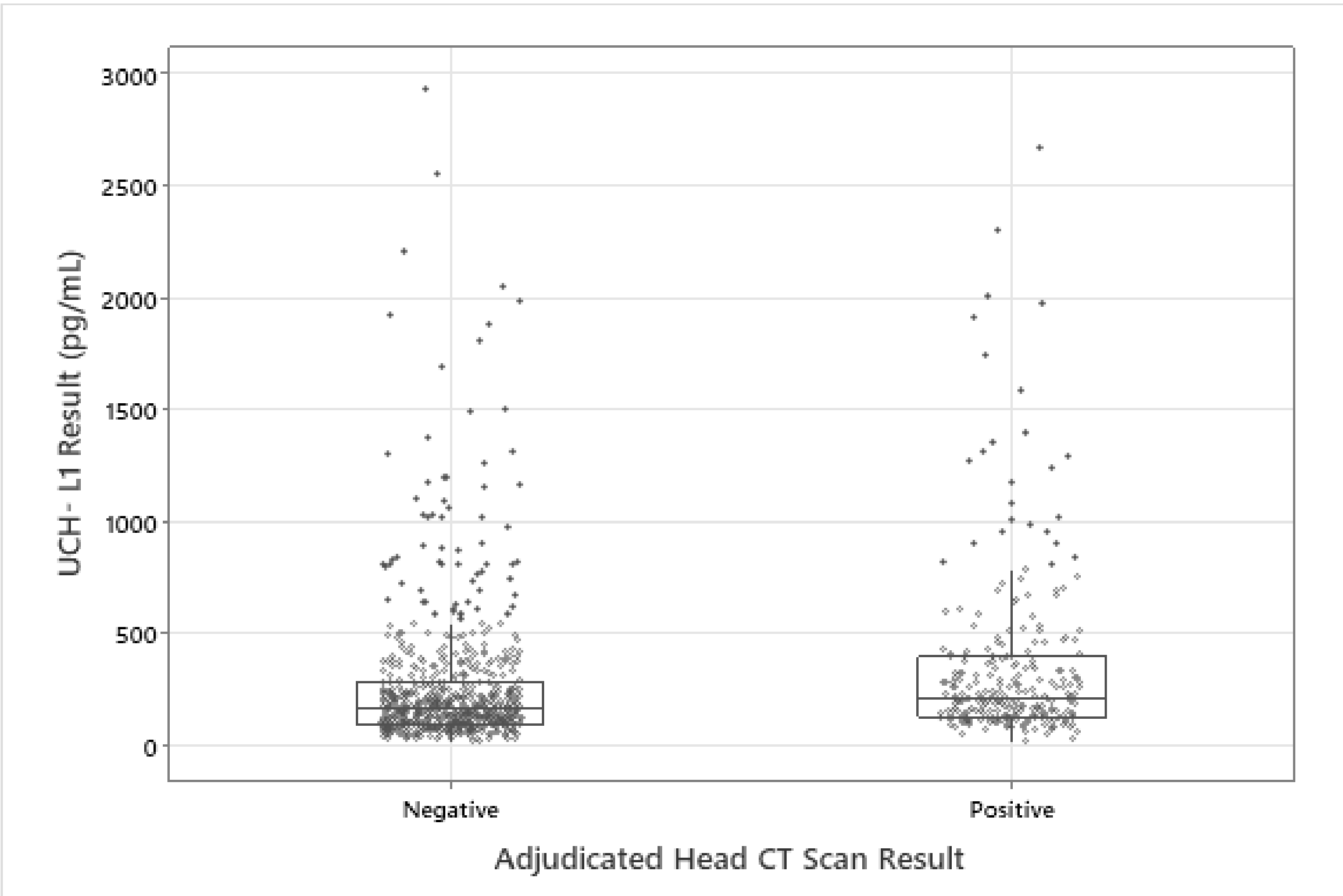

Abbott

en

i-STAT TBI Cartridge
03S09-25
B3S090
793051-01C

i-STAT TBI Cartridge

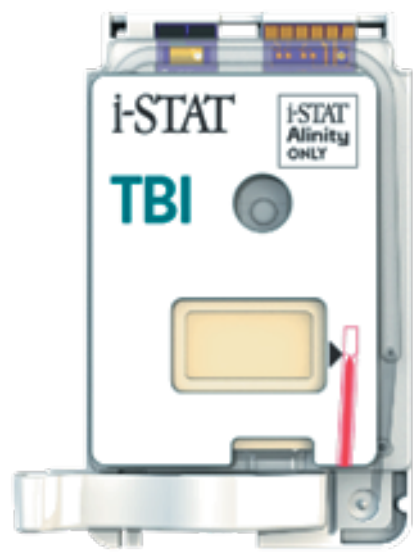

NAME

i-STAT TBI Cartridge (REF 03S09-25)

INTENDED USE

The i-STAT TBI test is a panel of *in vitro* diagnostic immunoassays for the quantitative measurements of glial fibrillary acidic protein (GFAP) and ubiquitin carboxyl-terminal hydrolase L1 (UCH-L1) in whole blood and a semi-quantitative interpretation of test results derived from these measurements, using the i-STAT Alinity instrument. The interpretation of test results is used, in conjunction with other clinical information, to aid in the evaluation of patients, 18 years of age or older, presenting with suspected mild traumatic brain injury (Glasgow Coma Scale score 13-15), which may include one of the following four clinical criteria: 1) any period of loss of consciousness, 2) any loss of memory for events immediately before and after the accident, 3) any alteration in mental state at the time of accident, and/or 4) focal neurological deficits, within 24 hours of injury, to assist in determining the need for a CT (computed tomography) scan of the head. A ‘Not Elevated’ test interpretation is associated with the absence of acute traumatic intracranial lesions visualized on a head CT scan.

The test is to be used with venous whole blood collected with EDTA anticoagulant in point of care or clinical laboratory settings by a healthcare professional.

SUMMARY AND EXPLANATION / CLINICAL SIGNIFICANCE

Test Principle

The i-STAT TBI cartridge is a multiplex immunoassay that contains assays for both glial fibrillary acidic protein (GFAP) and ubiquitin carboxyl-terminal hydrolase L1 (UCH-L1). The assays test for the presence of these biomarkers in a single whole blood sample and yield a semi-quantitative test interpretation based on measurements of both GFAP and UCH-L1 in approximately 15 minutes. The i-STAT TBI cartridge is designed to be run only on the i-STAT Alinity instrument.

Both assays on the cartridge use the sandwich enzyme-linked immunosorbent assay (ELISA) method with electrochemical detection of the resulting enzyme signal. The capture antibodies specific for the antigens (GFAP and UCH-L1) are each immobilized to a separate electrochemical sensor fabricated on a silicon chip. Also deposited in another location on the sensor silicon chip are the detection antibodies conjugated to the alkaline phosphatase enzyme (detection antibody-AP conjugates) that are specific to a separate region or epitope of each antigen. The whole blood sample is brought into contact with the sensors allowing the detection antibody-AP conjugates to dissolve into the sample. The antigens present in the sample interact with both the detection antibody-AP conjugates and the immobilized capture antibodies to form a sandwich (detection antibody-AP/antigen/capture antibody) on the

surfaces of their respective electrochemical sensors during an incubation period of approximately twelve minutes. The sample and excess detection antibody-AP conjugates are then washed off the sensors. Within the wash fluid is a substrate for the AP enzyme. The AP enzyme within the sandwich cleaves the substrate, releasing an electrochemically detectable product. The electrochemical (amperometric) sensor for each assay measures this enzyme product, which is proportional to the concentration of GFAP and UCH-L1 within the sample.

The i-STAT TBI cartridge is a single use test cartridge. The cartridge contains a biosensor chip and all reagents required to execute the test cycle. All fluid movements (test sample or reagent) are automatically controlled by the i-STAT Alinity instrument by electro-mechanical interaction with the cartridge. No additional reagents or steps are required to run the cartridge.

Clinical Significance

Traumatic brain injury (TBI) is the structural injury or physiologic disruption of brain function caused by the impact of an external mechanical force on the brain. The resulting injury can be ranked from mild to severe based on clinical symptoms, level of consciousness, and neuroimaging techniques. While severe TBI presents with more overt symptoms, patients presenting with mild TBI remain difficult to diagnose objectively. Computed tomography (CT), the most commonly used neuroimaging technique in the acute assessment of head injury patients, has advantages over Magnetic Resonance Imaging (MRI) due to its rapid acquisition and high spatial resolution for detailed anatomical structures in the head. An estimated 90% of head CT scans in patients suspected of having mild TBI have negative results for clinically important brain injuries [1]. A single non-contrast CT scan of the head exposes a patient to a dose of radiation comparable to eight months of background radiation [2]. Preventing unnecessary use of neuroimaging and associated radiation exposure is important in patient care, especially for preventing development of cataracts or malignant tumors involving radiosensitive organs such as the salivary gland, thyroid gland, and retina. Measurement of glial fibrillary acid protein (GFAP) and ubiquitin carboxyl-terminal hydrolase L1 (UCH-L1) released from the brain into the blood has been proposed as a method of reducing unnecessary radiation exposure in patients suspected of having mild TBI and provide an opportunity to improve the care of this patient group [3,4].

Glial Fibrillary Acid Protein

Glial fibrillary acidic protein (GFAP) is an astrocyte structural protein. GFAP is found in brain parenchyma. Metting and colleagues demonstrated that serum GFAP was increased in TBI patients with an abnormal CT and also demonstrated that GFAP was elevated in patients with axonal injury on MRI three months post injury [5]. In a study by Papa and colleagues, GFAP was detectable in serum less than 1 hour after head injury and it was able to reliably distinguish between trauma patients with mild TBI and those without head injury [6]. In this same study, blood GFAP levels were elevated in patients with traumatic intracranial abnormalities on CT compared with those without lesions and could also be used to predict those patients who required neurosurgical intervention [6].

Ubiquitin Carboxyl-Terminal Hydrolase L1

Ubiquitin carboxyl-terminal hydrolase-L1 (UCH-L1) is a protein that is involved in the metabolism of ubiquitin within neurons [7]. Increases in blood UCH-L1 have been detected in the serum of mild and moderate TBI patients within an hour of injury[8]. Levels measured within 4 hours of injury were significantly higher in those with TBI lesions on CT than those with a normal intracranial appearance on CT. Blood levels of UCH-L1 have been demonstrated to be able to discriminate mild TBI patients from patients without head injuries and, similar to GFAP, UCH-L1 levels were much higher in patients who required neurosurgical intervention [8].

REAGENTS

Contents

Each i-STAT TBI cartridge contains all the necessary reagents needed to perform the test. The cartridge contains a buffer and preservatives. A list of reactive ingredients is provided below:

Reactive Ingredient	Biological Source	Minimum Quantity
Antibody / Alkaline Phosphatase Conjugate	Murine IgG / Bovine intestine	0.005 µg
IgG	Murine IgG	18.0 µg
IgG	Caprine IgG	12.0 µg
IgG	Leporine IgG	18.0 µg
IgM	Murine IgM	0.60 µg
Sodium Aminophenyl Phosphate	N/A	3 mg
Heparin	Porcine intestine	0.5 IU

Warnings and Precautions

- For *in vitro* diagnostic use.
- DO NOT REUSE—cartridges are intended for single-use only.
- Although the sample is contained within the cartridge, used cartridges should be disposed of as biohazardous waste according to local, state, and national regulatory guidelines.
- The i-STAT System automatically runs a comprehensive set of quality checks of instrument and cartridge performance each time a sample is tested. This internal quality system will suppress results by generating a Quality Check Failure (QCF), if the instrument or cartridge does not meet certain specifications. To minimize the probability of delivering a result with medically significant error the internal specifications are very stringent. It is typical for the system to suppress a very small percentage of results in normal operation given the stringency of these specifications. If however the instrument or cartridges have been compromised, results may be persistently suppressed, and one or the other must be replaced to restore normal operating conditions. Where unavailability of results while awaiting replacement of instruments or cartridges is unacceptable, Abbott Point of Care Inc. recommends maintaining both a backup i-STAT Alinity instrument and cartridges from an alternate lot number.
- When a QCF occurs, a code number and the next step to be taken will be displayed on the i-STAT instrument. Refer to the i-STAT Alinity System Operations Manual for additional information on QCFs. The failure rate due to QCFs may be as high as 3.41%. The rate of failure for two consecutive cartridges due to QCFs may be as high as 0.34%.

For additional warnings and precautions about the i-STAT Alinity System refer to the i-STAT Alinity System Operations Manual located at www.globalpointofcare.abbott.

Storage Conditions

Note: For optimal performance, cartridge storage at 2 to 8 °C (35 to 46 °F) is recommended.

- The expiration date, expressed as YYYY-MM-DD on the packaging, indicates the last day the product may be used.
- Refrigerated at 2 to 8 °C (35 to 46 °F) until expiration date.
- Room Temperature at 18 to 30 °C (64 to 86 °F) for up to 14 days.

- Allow refrigerated cartridges to equilibrate at room temperature for 5 minutes for a single cartridge and 1 hour for an entire box before use as described in Procedure for Cartridge Testing below. Cartridges must be at room temperature before removing from the portion pack.

INSTRUMENTS

The i-STAT TBI cartridge is intended for use with the i-STAT Alinity instrument.

For a detailed description of the instrument and system procedures, refer to the i-STAT Alinity System Operations Manual located at www.globalpointofcare.abbott.

SPECIMEN COLLECTION AND PREPARATION FOR ANALYSIS

Specimen Types

Venous whole blood collected with EDTA anticoagulant

Sample volume: 20 µL

Blood Collection Options and Test Timing (time from collection to cartridge fill)

Assay	Evacuated Tubes	Test Timing
GFAP<br>UCH-L1	EDTA<br>Fill tube according to manufacturer's recommendation	1 hour

PROCEDURE FOR CARTRIDGE TESTING

The i-STAT System should be used by healthcare professionals trained and certified to use the system and should be used according to the facility's policies and procedures.

Each cartridge is sealed in a portion pack (individual cartridge package) for protection during storage--do not use if the portion pack has been damaged or punctured.

- A cartridge should not be removed from its protective portion pack until it is at room temperature (18-30 °C or 64-86 °F). For best results, the cartridge and instrument should be at room temperature.
- Since condensation on a cold cartridge may prevent proper contact with the instrument allow refrigerated cartridges to equilibrate at room temperature for 5 minutes for a single cartridge and 1 hour for an entire box before use.
- Use a cartridge immediately after removing it from its protective portion pack; prolonged exposure may cause a cartridge to fail a Quality Check.
- Do not return unopened, previously refrigerated cartridges to the refrigerator.
- Cartridges may be stored at room temperature for the time frame indicated on the cartridge box.

Performing Patient Analysis

1. Press the power button to turn instrument on.
2. From the Home screen, touch *Perform Patient Test.* This initiates the patient testing pathway.

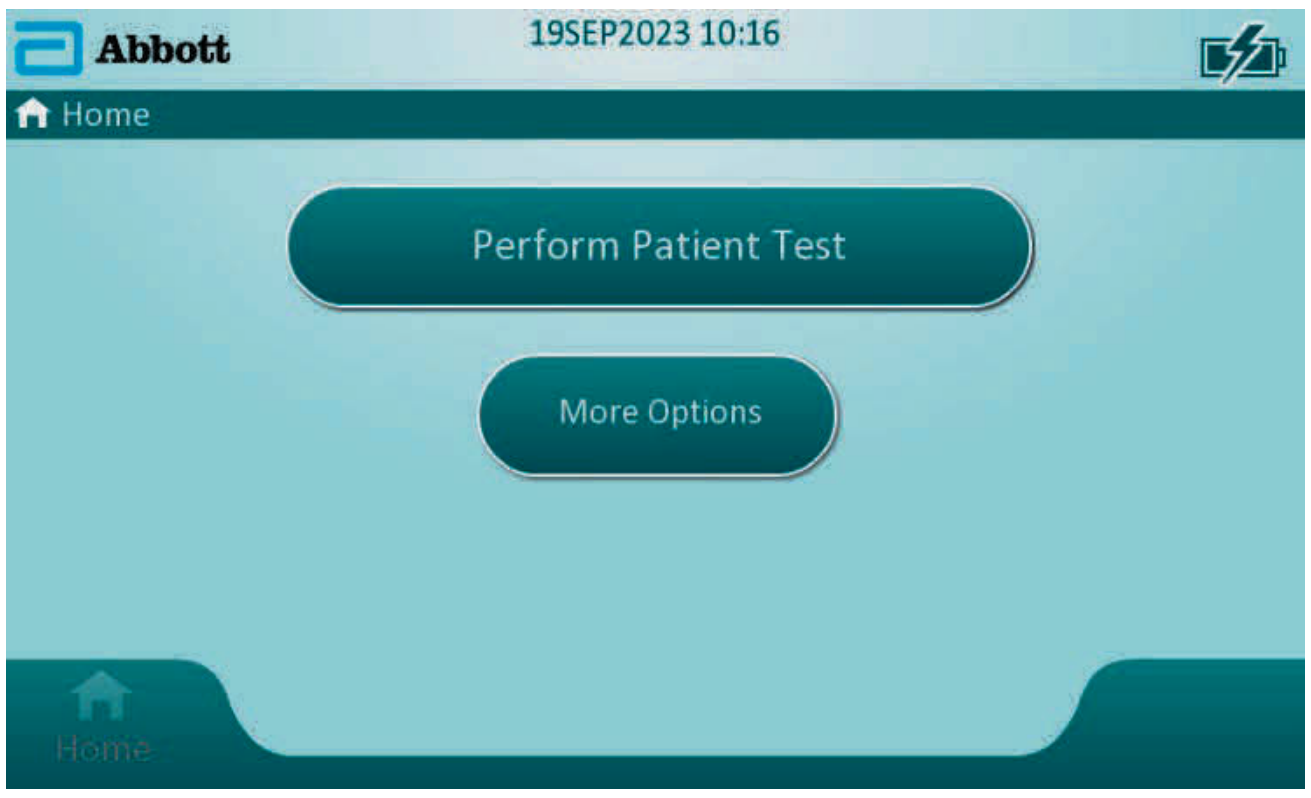

3. Follow instructions on the screen to “Scan or Enter OPERATOR ID”.
4. Follow instructions on the screen to “Scan or Enter PATIENT ID”
5. Continue to follow prompts on the screen to proceed with patient testing. “Scan (CARTRIDGE POUCH) Barcode”, Scanning is required. Information cannot be entered manually.
6. Follow instructions on the screen to “Close and Insert Filled Cartridge”. The action buttons at the bottom of the screen allow forward, backward and pause functionality.

Filling and Sealing the Cartridge

7. Place the room temperature equilibrated cartridge on a flat surface.
8. Invert the EDTA blood collection tube at least 10 times. Remove a small sample from the EDTA tube.
9. Fill the cartridge immediately by directing the tip of the transfer device into the sample well of the cartridge.
10. Slowly dispense sample until the sample reaches the ‘fill to’ mark indicated on the cartridge. Cartridge is properly filled when the sample reaches the ‘fill to’ mark and a small amount of sample is in the sample well. The sample should be continuous, no bubbles or breaks.
11. Slide the closure clip of the cartridge over the sample well.
12. **Immediately** insert the sealed cartridge into the cartridge port until it clicks into place. Once the cartridge is inserted, “Contacting Cartridge” will display followed by the countdown bar. The following alerts are also displayed: “Cartridge locked in instrument. Do not attempt to remove the Cartridge” and “Testing – Instrument Must Remain Level”.
13. Wait for the test to complete. When the test is complete, the results are displayed.

Analysis Time

15 minutes.

Results

The i-STAT TBI test is a semi-quantitative assay.

Interpretation of Results

i-STAT TBI results are displayed on two pages on the i-STAT Alinity instrument. The first page is the test interpretation (Elevated, Not Elevated, Repeat Test) shown in the table below. The second page shows the quantitative results. In the case of a Repeat Test interpretation, the second page is not available. An example of the interpretation and results pages are below.

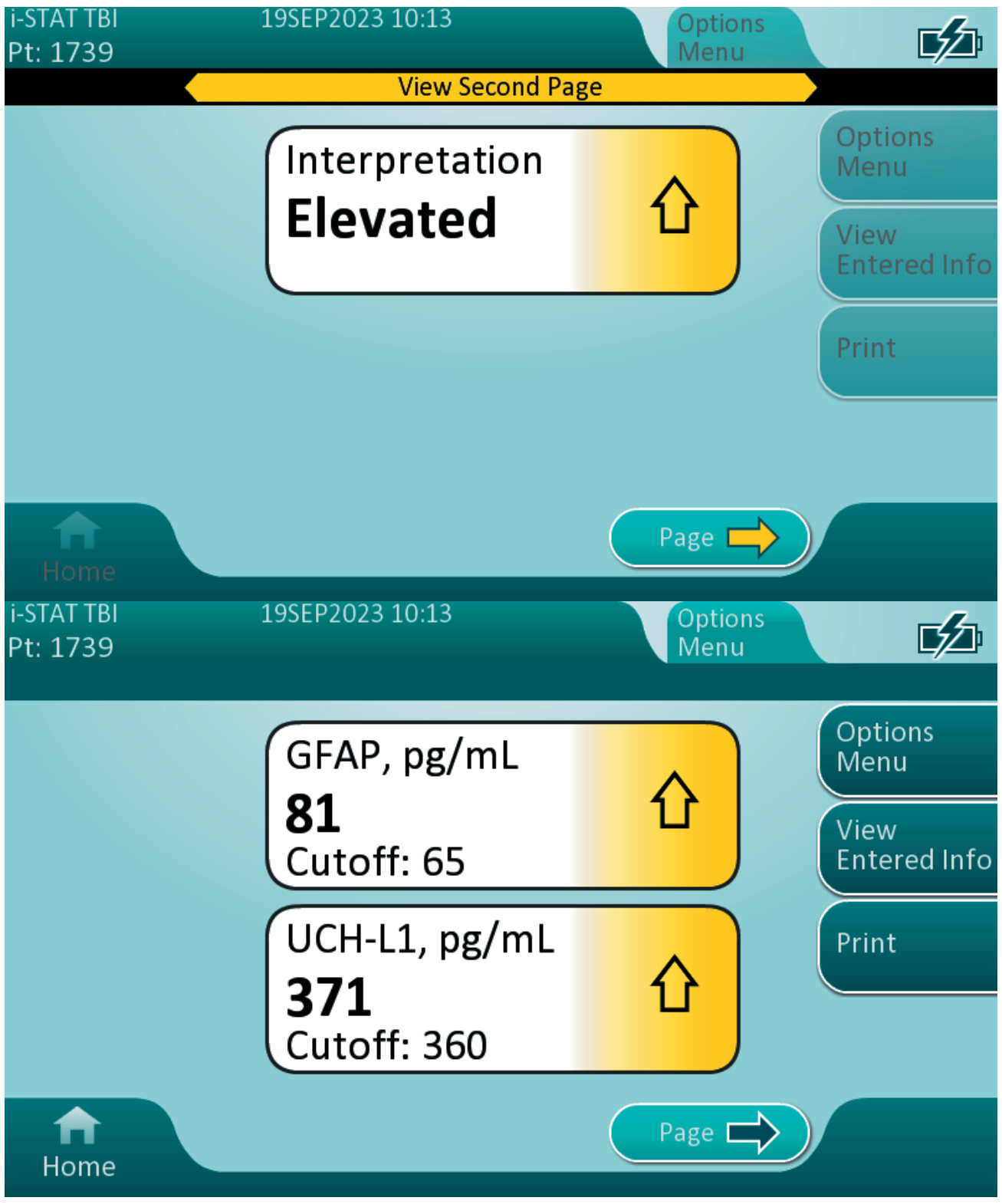

- In the example, the result bubbles are marked with yellow. On the interpretation page, yellow indicates an "elevated" interpretation. On the results page, yellow indicates quantitative results above cutoff. This is intended to draw the attention of the operator.
- The blinking page button at the bottom of the screen appears when there is more than one page of results. All action tabs are inactive until the second page of results has been viewed.
- An audible cue will be heard when results are ready. Touch **Silence** or remove cartridge to silence.

The table below outlines the test interpretation matrix based on the GFAP and UCH-L1 assay results relative to cutoffs. The assay cutoffs were established to be 65 pg/mL for GFAP and 360 pg/mL for UCH-L1.

Test Interpretation Matrix

GFAP Assay Result (relative to cutoff of 65 pg/mL)	UCH-L1 Assay Result (relative to cutoff of 360 pg/mL)	Test Interpretation
Below	Below	Not Elevated
Below	Equal or Above	Elevated
Equal or Above	Below	Elevated
Equal or Above	Equal or Above	Elevated
Equal or Above	***[†]	Elevated
Below	Not reported	Repeat Test[‡]
***[†]	Equal or Above	Elevated
Not reported	Below	Repeat Test[‡]
Not reported	Not reported	Repeat Test[‡]

[†] Starout condition. "***" is displayed rather than a quantitative result. Instrument is unable to determine a quantitative result from a particular sensor on the cartridge due to detection of a signal from the sensor that is uncharacteristic. Because the other assay provides a result at or above cutoff, a test interpretation can be reported. Refer to the i-STAT Alinity System Operations Manual for addition information on starouts.

[‡] Results are not available for both assays, or for one assay and the other assay provides a result below cutoff. Repeat Test will appear as a Quality Check Failure (QCF) screen with error code 152-01. Repeat test with a freshly filled cartridge. If the same QCF displays, contact the system administrator for further instruction. Refer to the i-STAT Alinity System Operations Manual for additional information on QCFs.

'Not Elevated' test interpretation is associated with the absence of acute traumatic intracranial lesions visualized on a head CT scan.

'Elevated' test interpretation suggests further evaluation by head CT scan should be considered.

REPORTABLE RANGE

Assay	Lower Limit of Reportable Range (pg/mL)	Upper Limit of Reportable Range (pg/mL)
GFAP	47	10000
UCH-L1	87	3200

Results are preceded by the symbols for greater than (>) or less than (<) if the result is outside of the reportable range.

PROCEDURE FOR QUALITY TESTING

Quality Control

For information on performing liquid quality control, refer to the i-STAT TBI Control Levels 1, 2 instructions for use located at www.globalpointofcare.abbott.

Calibration Verification

For information on performing calibration verification testing, refer to the i-STAT TBI Calibration Verification Levels 1-3 instructions for use located at www.globalpointofcare.abbott.

Each laboratory should follow local, state and national regulations regarding quality control materials.

METROLOGICAL TRACEABILITY

The i-STAT System test for glial fibrillary acidic protein (GFAP) or ubiquitin carboxyl-terminal hydrolase L1 (UCH-L1) measures GFAP and UCH-L1 amount-of-substance concentration in the plasma fraction of whole blood (units of measure: pg/mL) for *in vitro* diagnostic use.

There are no internationally recognized standard reference materials available for either glial fibrillary acidic protein (GFAP) or ubiquitin carboxyl-terminal hydrolase L1 (UCH-L1). GFAP and UCH-L1 values assigned to i-STAT controls and calibration verification materials are traceable to Abbott's working calibrators prepared using recombinant GFAP and UCH-L1 (expressed and purified from *E. coli*). The working calibrators are traceable to an in-house Reference Standard prepared from recombinant GFAP and UCH-L1 (expressed and purified from *E. coli*).

i-STAT System controls and calibration verification materials are validated for use only with the i-STAT System and assigned values may not be commutable with other methods. Further information regarding metrological traceability is available from Abbott Point of Care Inc.

To obtain additional information and technical support, refer to the company website at www.globalpointofcare.abbott.

EXPECTED VALUES

A reference interval study was conducted in accordance with CLSI EP28-A3c[9], with a US-based general population. Venous whole blood specimens from 150 subjects between the ages of 18 and 83 years self-reporting no history of neurological disease within 1 year were tested with the i-STAT TBI cartridge with the i-STAT Alinity System to determine GFAP and UCH-L1 levels. Based on the test results, a 95% reference interval of an apparently healthy population of each biomarker was determined to be <47 – 53 pg/mL for GFAP and <87 – 251 pg/mL UCH-L1.

The proportion of GFAP and UCH-L1 measurements that were below the lower limit of the assay range was 94.0% (141/150) and 56.7% (85/150), respectively. Based on the test results with the i-STAT TBI cartridge with the i-STAT Alinity System, 0.7% (1/150) of the individuals from an apparently healthy population had a test interpretation of "elevated" for biomarkers.

CLINICAL PERFORMANCE

A pivotal study using prospectively collected venous whole blood specimens was conducted to establish the clinical performance of the i-STAT TBI test. The testing of the whole blood specimens was conducted at twenty clinical sites in the United States. The facilities used and the study staff that performed the testing were representative of point of care (POC) end-users. Study staff that performed testing included medical practitioners, nurses, study research coordinators, research assistants, research associates, laboratory assistants and phlebotomists.

The specimens were collected in a prospective, multi-center clinical study that enrolled consenting men and women 18 years of age or older who presented to emergency departments (ED) with suspected traumatic brain injury with initial Glasgow Coma Scale (GCS) scores of 13-15 and who had a computed tomography (CT) scan ordered as part of their standard clinical care. Subjects were enrolled at 20 clinical sites in the United States.

CT scans were performed in accordance with the clinical site's standard of care. Images were transmitted to a central data capture system. Images were interpreted by at least two neuroradiologists who were masked to other clinical and laboratory data; procedures for scoring

images were established before conducting image review. The clinical outcome was based on the consensus interpretation between two neuroradiologists with adjudication by a third neuroradiologist if necessary. Outcomes were positive or negative as defined by the presence or absence of acute traumatic intracranial lesions, respectively. Acute intracranial lesion was defined as any trauma induced or related finding visualized upon head CT scan.

Venous whole blood was collected into K_2EDTA blood collection tubes from each subject within 24 hours of head injury using venipuncture. Specimens from 970 subjects were included in the analysis.

The demographic characteristics of the subjects represented in the clinical performance analysis are summarized in the table below.

Demographic Characteristics

Characteristic	Head CT Scan Result		Total
	Positive	Negative	
**N**	283	687	970
**Age (Years)**			
Mean	51.1	45.0	46.8
Median	52.0	42.0	46.0
Standard Deviation	19.68	18.92	19.33
Minimum	18	18	18
Maximum	96	97	97
**Gender, N (%)**			
Male	187 (66.1%)	434 (63.2%)	621 (64.0%)
Female	94 (33.2%)	252 (36.7%)	346 (35.7%)
Unspecified/ Not Reported	2 (0.7%)	1 (0.1%)	3 (0.3%)
**Race, N (%)**			
White	224 (79.2%)	441 (64.2%)	665 (68.6%)
Black or African American	20 (7.1%)	152 (22.1%)	172 (17.7%)
Asian	11 (3.9%)	38 (5.5%)	49 (5.1%)
Native Hawaiian/Pacific Islander	4 (1.4%)	6 (0.9%)	10 (1.0%)
American Indian or Alaska Native	4 (1.4%)	8 (1.2%)	12 (1.2%)
Asian, White	2 (0.7%)	3 (0.4%)	5 (0.5%)
Asian, Black or African American	0 (0.0%)	1 (0.1%)	1 (0.1%)
Black or African American, American Indian or Alaska Native	0 (0.0%)	2 (0.3%)	2 (0.2%)
White, Black or African American	0 (0.0%)	5 (0.7%)	5 (0.5%)
Not Reported	10 (3.5%)	19 (2.8%)	29 (3.0%)
Unknown	8 (2.8%)	12 (1.7%)	20 (2.1%)
**Ethnicity, N (%)**			
Hispanic or Latino	67 (23.7%)	121 (17.6%)	188 (19.4%)
Not Hispanic or Latino	209 (73.9%)	551 (80.2%)	760 (78.4%)

Characteristic	Head CT Scan Result		Total
	Positive	Negative	
Unknown	6 (2.1%)	6 (0.9%)	12 (1.2%)
Not Reported	1 (0.4%)	9 (1.3%)	10 (1.0%)

The head injury characteristics of the 970 subjects in the performance analysis were tabulated. Information regarding time from head injury to exam, head injury to CT scan, and head injury to blood draw, as well as GCS, neurological assessment, mechanism of injury, and physical evidence of trauma, categorized by head CT scan results, are shown in the table below.

Head Injury Characteristics

Assessment	Head CT Scan Result		Total
	Positive	Negative	
**N**	283	687	970
**Time from head injury to Initial Assessment (hours)***			
Mean	2.0	1.3	1.5
Median	1.0	0.8	0.9
Standard Deviation	2.01	1.45	1.67
Range	(1.0, 10.2)	(0.8, 10.0)	(0.8, 10.2)
**Time from head injury to CT scan (hours)***			
Mean	2.6	2.5	2.6
Median	1.7	2.0	1.9
Standard Deviation	2.37	1.80	1.98
Range	(0.2, 11.4)	(0.3, 10.7)	(0.2, 11.4)
**Time from head injury to blood draw (hours)***			
Mean	14.5	8.8	10.4
Median	13.5	5.8	8.1
Standard Deviation	6.65	6.43	6.99
Range	(2.0, 24.0)	(1.5, 24.0)	(1.5, 24.0)
**Glasgow Coma Score – N (%)**			
13	28 (9.9%)	11 (1.6%)	39 (4.0%)
14	79 (27.9%)	90 (13.1%)	169 (17.4%)
15	176 (62.2%)	586 (85.3%)	762 (78.6%)
**Neurological assessment - N (%) of subjects experiencing:**			
Loss of Consciousness (LOC)	225 (79.5%)	450 (65.5%)	675 (69.6%)
Confusion/Alteration of Consciousness (AOC)	195 (68.9%)	504 (73.4%)	699 (72.1%)
Vomiting	24 (8.5%)	21 (3.1%)	45 (4.6%)

Assessment	Head CT Scan Result		Total
	Positive	Negative	
Post Traumatic Amnesia (PTA)	196 (69.3%)	409 (59.5%)	605 (62.4%)
Post Traumatic Seizures	3 (1.1%)	0 (0.0%)	3 (0.3%)
Subjects with Drug Intoxication at the Time of Presentation to Facility	48 (17.0%)	66 (9.6%)	114 (11.8%)
Subjects with Alcohol Intoxication at the Time of Presentation to Facility	49 (17.3%)	61 (8.9%)	110 (11.3%)
**Mechanism of Injury - N (%) of subjects affected:**			
Acceleration/Deceleration	68 (24.0%)	221 (32.2%)	289 (29.8%)
Direct Impact (blow to head)	44 (15.5%)	85 (12.4%)	129 (13.3%)
Direct Impact (head against object)	157 (55.5%)	437 (63.6%)	594 (61.2%)
Crush	0 (0.0%)	3 (0.4%)	3 (0.3%)
Blast	0 (0.0%)	1 (0.1%)	1 (0.1%)
Ground level fall	82 (29.0%)	170 (24.7%)	252 (26.0%)
Fall from Height > 1 meter (3 feet)	39 (13.8%)	79 (11.5%)	118 (12.2%)
Other	15 (2.2%)	7 (2.5%)	22 (2.3%)
**Physical Evidence* – N (%) of subjects with:**			
Visible Trauma Above Clavicle	214 (75.6%)	422 (61.4%)	636 (65.6%)
Signs of Basal Skull Fracture	37 (13.1%)	7 (1.0%)	44 (4.5%)

*Based on time subject arrived at the study hospital for neurological assessments.

The i-STAT TBI test clinical performance estimates are shown in the table below. Of the 970 subjects, 283 subjects had positive CT scan results. Of these 283 subjects, 273 had an ‘elevated’ i-STAT TBI test result with venous whole blood specimens (clinical sensitivity = 96.5% (273/283)). Ten subjects with CT scan positive results had an i-STAT TBI test result of ‘not elevated’. The rate of false negative (FN) results was 3.5% (10/283). The ten subjects identified as false negative using the i-STAT TBI test for the venous whole blood specimens were not identified with lesion requiring surgical intervention. 14 subjects were associated with lesions requiring surgical intervention and the i-STAT TBI test correctly classified all 14 CT-positive subjects with a test result of ‘elevated.’ Of the 687 subjects with negative CT scan results, 277 had an i-STAT TBI test result of ‘not elevated’ (clinical specificity = 40.3% (277/687)). The rate of False Positive (FP) results was 59.6% (410/687).

In the clinical study, the prevalence of adjudicated CT scan positive subjects was 29.2% (283/970). Overall, there were 287 subjects with i-STAT TBI test results of ‘not elevated’. Of these, 277 subjects had negative CT scan results. The Negative Predictive Value (NPV) of the assay was 96.5% (277/287) for prevalence of 29.2%. The value of the NPV at 6% prevalence is 99.4% (95% CI: 99.0%, 99.7%).

Clinical Performance

i-STAT TBI Test Interpretation	Adjudicated CT Scan Result Positive	Negative	Total
**Elevated**	273	410	683
**Not Elevated**	10	277	287
**Total**	283	687	970

Clinical Performance Parameters	N=970	95% Confidence Interval
**Prevalence of CT Positive Subjects**	29.2% (283/970)	N/A
**Clinical Sensitivity**	96.5% (273/283)	(93.6%, 98.1%)*
**Clinical Specificity**	40.3% (277/687)	(36.7%, 44.0%)*
**Negative Predictive Value (NPV)**	96.5% (277/287)	(93.7%, 98.1%)†
**Positive Predictive Value (PPV)**	40.0% (273/683)	(38.4%, 41.5%)†
**Likelihood Ratio Negative (LRN)**	0.09	(0.05, 0.16)‡
**Likelihood Ratio Positive (LRP)**	1.62	(1.52, 1.73)‡

*95% confidence intervals are calculated using the Wilson score method for a binomial portion (see CLSI EP12-Ed3)
†95% confidence intervals for predictive values are calculated based on the confidence intervals of the corresponding likelihood ratios
‡95% confidence intervals are calculated using asymptotic method for a ratio of two binomial proportions

PERFORMANCE CHARACTERISTICS

The typical performance of GFAP and UCH-L1 assays within the i-STAT TBI cartridge with i-STAT Alinity System are summarized below.

Precision

A study was conducted in point-of-care settings at three (3) clinical sites based on CLSI guideline EP05-A3 [10] . Venous whole blood samples representing GFAP and UCH-L1 concentrations spanning the reportable range were prepared at each site (eight (8) GFAP and eight (8) UCH-L1 samples at Site 1; eight (8) GFAP and 13 UCH-L1 samples at Site 2; seven (7) GFAP and eight (8) UCH-L1 samples at Site 3). At each site, each sample was tested on the same day by two (2) operators with three (3) runs and four (4) assay measurements per run for a total of 24 measurements per sample per site. For each sample at each site, the repeatability, between-instrument, between-operator, and within-site imprecision of the GFAP and UCH-L1 assays were calculated and are shown in the tables below. Within-site imprecision includes the repeatability, between-instrument, and between-operator components of imprecision.

GFAP Assay Precision in Whole Blood at Point-of-Care Site 1

Sample	N	Mean	Repeatability SD (pg/mL)	Repeatability CV (%)	Between-Instrument SD (pg/mL)	Between-Instrument CV (%)	Between-Operator SD (pg/mL)	Between-Operator CV (%)	Within-Site SD (pg/mL)	Within-Site CV (%)
1	24	63.3	9.84	15.53	0.00	0.00	2.98	4.70	10.28	16.23
2	23‡	64.3	11.72	18.23	6.76	10.51	4.25	6.61	14.18	22.06
3	24	103.5	10.85	10.48	0.00	0.00	2.22	2.14	11.07	10.70
4	23‡	128.5	14.51	11.29	0.00	0.00	0.00	0.00	14.51	11.29
5	24	986.3	88.48	8.97	0.00	0.00	0.00	0.00	88.48	8.97
6	24	3431.6	338.46	9.86	0.00	0.00	104.36	3.04	354.19	10.32
7	24	6371.3	637.41	10.00	0.00	0.00	162.96	2.56	657.91	10.33
8	24	7836.9	730.91	9.33	0.00	0.00	102.96	1.31	738.13	9.42

GFAP Assay Precision in Whole Blood at Point-of-Care Site 2

Sample	N	Mean	Repeatability SD (pg/mL)	Repeatability CV (%)	Between-Instrument SD (pg/mL)	Between-Instrument CV (%)	Between-Operator SD (pg/mL)	Between-Operator CV (%)	Within-Site SD (pg/mL)	Within-Site CV (%)
1	24	60.9	11.08	18.18	0.00	0.00	2.15	3.53	11.28	18.52
2	24	57.7	7.24	12.56	4.60	7.97	5.28	9.16	10.07	17.47
3	24	148.1	12.08	8.16	0.00	0.00	0.00	0.00	12.08	8.16
4	24	83.7	6.98	8.34	0.00	0.00	0.00	0.00	6.98	8.34
5	24	900.6	28.89	3.21	10.84	1.20	0.00	0.00	30.85	3.43
6	24	3731.1	161.63	4.33	0.00	0.00	121.29	3.25	202.08	5.42
7	24	5762.3	289.18	5.02	0.00	0.00	0.00	0.00	289.18	5.02
8	24	8310.3	499.50	6.01	0.00	0.00	0.00	0.00	499.50	6.01

GFAP Assay Precision in Whole Blood at Point-of-Care Site 3

Sample	N	Mean	Repeatability SD (pg/mL)	Repeatability CV (%)	Between-Instrument SD (pg/mL)	Between-Instrument CV (%)	Between-Operator SD (pg/mL)	Between-Operator CV (%)	Within-Site SD (pg/mL)	Within-Site CV (%)
1	23‡	58.9	4.47	7.59	2.60	4.41	0.00	0.00	5.17	8.78
2	22§	67.2	16.54	24.62	0.00	0.00	0.00	0.00	16.54	24.62
3	24	145.4	10.54	7.25	0.00	0.00	3.28	2.26	11.03	7.59
4	24	962.1	56.81	5.90	24.53	2.55	0.00	0.00	61.88	6.43
5	24	2954.5	167.36	5.66	0.00	0.00	3.12	0.11	167.39	5.67
6	24	6226.4	246.48	3.96	18.23	0.29	20.69	0.33	248.02	3.98
7	23¶	8366.9	502.57	6.01	0.00	0.00	168.21	2.01	529.97	6.33

‡ one (1) result not obtained due to a quality check failure (QCF) or star-out error
§ two (2) results not obtained due to a quality check failure (QCF) or star-out error
¶ one (1) result not obtained due to operator error

UCH-L1 Assay Precision in Whole Blood at Point-of-Care Site 1

Sample	N	Mean	Repeatability SD (pg/mL)	Repeatability CV (%)	Between-Instrument SD (pg/mL)	Between-Instrument CV (%)	Between-Operator SD (pg/mL)	Between-Operator CV (%)	Within-Site SD (pg/mL)	Within-Site CV (%)
1	23‡	215.7	16.58	7.69	0.00	0.00	0.00	0.00	16.58	7.69
2	24	243.5	19.35	7.95	0.00	0.00	11.14	4.57	22.33	9.17
3	24	333.7	28.69	8.60	12.79	3.83	17.15	5.14	35.79	10.7
4	22	438.9	55.79	12.71	0.00	0.00	0.00	0.00	55.79	12.7
5	24	486.7	25.57	5.25	6.37	1.31	9.33	1.92	27.96	5.74
6	24	1451.4	106.30	7.32	0.00	0.00	70.00	4.82	127.28	8.77
7	24	1746.3	96.10	5.50	0.00	0.00	0.00	0.00	96.10	5.50
8	22‡‖	3020.3	146.12	4.84	20.19	0.67	57.99	1.92	158.50	5.25

UCH-L1 Assay Precision in Whole Blood at Point-of-Care Site 2

Sample	N	Mean	Repeatability SD (pg/mL)	Repeatability CV (%)	Between-Instrument SD (pg/mL)	Between-Instrument CV (%)	Between-Operator SD (pg/mL)	Between-Operator CV (%)	Within-Site SD (pg/mL)	Within-Site CV (%)
1	24	183.0	15.28	8.35	2.89	1.58	0.00	0.00	15.55	8.50
2	24	220.2	19.75	8.97	0.00	0.00	0.00	0.00	19.75	8.97
3	24	232.3	15.71	6.76	0.00	0.00	0.00	0.00	15.71	6.76
4	24	360.8	26.99	7.48	18.27	5.06	0.00	0.00	32.59	9.03
5	24	413.0	38.18	9.25	10.68	2.59	5.52	1.34	40.03	9.69
6	24	535.1	61.35	11.47	0.00	0.00	10.79	2.02	62.29	11.64
7	23‡	630.5	49.93	7.92	0.00	0.00	5.97	0.95	50.29	7.98
8	24	675.0	50.54	7.49	20.74	3.07	0.00	0.00	54.63	8.09
9	23‡	935.1	62.83	6.72	20.06	2.15	0.00	0.00	65.95	7.05
10	21§	1114.1	59.38	5.33	0.00	0.00	0.00	0.00	59.38	5.33
11	23‡	2286.3	121.21	5.30	0.00	0.00	0.00	0.00	121.21	5.30

12	24	2319.1	139.38	6.01	42.48	1.83	66.90	6.91	160.34	6.91
13	21#	2945.8	141.67	4.81	0.00	0.00	0.00	0.00	141.67	4.81

UCH-L1 Assay Precision in Whole Blood at Point-of-Care Site 3

Sample	N	Mean	Repeatability SD (pg/mL)	Repeatability CV (%)	Between-Instrument SD (pg/mL)	Between-Instrument CV (%)	Between-Operator SD (pg/mL)	Between-Operator CV (%)	Within-Site SD (pg/mL)	Within-Site CV (%)
1	24	182.5	14.24	7.80	0.00	0.00	0.48	0.26	14.25	7.81
2	24	204.2	16.51	8.08	0.00	0.00	11.65	5.71	20.21	9.90
3	24	357.1	35.46	9.93	0.00	0.00	5.71	1.60	35.92	10.06
4	24	392.8	39.52	10.06	12.15	3.09	0.00	0.00	41.35	10.53
5	24	522.6	42.40	8.11	0.00	0.00	9.55	1.83	43.46	8.32
6	24	1213.4	54.20	4.47	34.69	2.86	0.00	0.00	64.35	5.30
7	24	1947.1	118.94	6.11	0.00	0.00	0.00	0.00	118.94	6.11
8	21‡‖¶	2829.4	150.88	5.33	59.80	2.11	81.45	2.88	181.59	6.42

‡ one (1) result not obtained due to a quality check failure (QCF) or star-out error

§ three (3) results not obtained due to a quality check failure (QCF) or star-out error

¶ one (1) result not obtained due to operator error

‖ one (1) result not measurable due to being above the measurement range

three (3) results not measurable due to being above the measurement range

Plasma and Controls Within-Laboratory Precision

Plasma samples representing five (5) concentrations of GFAP and six (6) concentrations of UCH-L1 spanning the reportable range as well as the i-STAT TBI Controls Level 1 (L1) and Level 2 (L2) were used to assess assay precision. A single-site study includes three (3) cartridge lots and was conducted based on CLSI guideline EP05-A3[10]. Each sample was tested for at least 20 days with two (2) runs per day and two (2) results per run for a total of 80 measurements per sample per cartridge lot. Runs were separated by a minimum of 2 hours.

The repeatability, between-run, between-day, and between-lot components of imprecision for the GFAP and UCH-L1 assays are shown in tables below.

GFAP Assay Precision

Sample	N	Mean (pg/mL)	Repeatability SD (pg/mL)	Repeatability CV (%)	Between-run SD (pg/mL)	Between-run CV (%)	Between-day SD (pg/mL)	Between-day CV (%)	Between-lot SD (pg/mL)	Between-lot CV (%)	Within-Laboratory SD (pg/mL)	Within-Laboratory CV (%)
1	240	78.8	3.04	3.86	0.85	1.07	0.57	0.72	2.17	2.76	3.90	4.95
2	240	98.6	6.03	6.12	1.40	1.42	0.72	0.73	2.57	2.61	6.78	6.87
3	240	880.6	21.29	2.42	15.78	1.79	1.66	0.19	9.76	1.11	28.79	3.27
4	240	4415.3	144.73	3.28	67.27	1.52	17.25	0.39	135.59	3.07	212.16	4.81
5	240	8346.7	285.03	3.41	151.07	1.81	56.69	0.68	347.63	4.16	479.49	5.74

UCH-L1 Assay Precision

Sample	N	Mean (pg/mL)	Repeatability SD (pg/mL)	Repeatability CV (%)	Between-run SD (pg/mL)	Between-run CV (%)	Between-day SD pg/mL	Between-day CV (%)	Between-lot SD (pg/mL)	Between-lot CV (%)	Within-Laboratory SD (pg/mL)	Within-Laboratory CV (%)
1	240	159.9	11.91	7.45	3.44	2.15	0.76	0.48	4.92	3.08	13.54	8.47
2	240	255.7	18.11	7.08	4.97	1.94	3.17	1.24	6.21	2.43	20.33	7.95
3	240	488.8	26.47	5.42	15.02	3.07	5.93	1.21	11.56	2.37	33.53	6.86
4	240	826.2	49.40	5.98	23.07	2.79	12.72	1.54	24.38	2.95	61.92	7.49
5	240	1763.7	100.60	5.70	26.56	1.51	29.57	1.68	84.62	4.80	138.92	7.88
6	240	2190.3	126.88	5.79	46.79	2.14	23.66	1.08	105.13	4.80	176.27	8.05

Precision performance observed with the i-STAT TBI Controls across 3 cartridge lots are tabulated in the table below.

GFAP and UCH-L1 assay Precision with i-STAT TBI Controls

Sample	N	Mean (pg/mL)	Repeatability		Between-run		Between-day		Between-lot		Within-Laboratory	
			SD (pg/mL)	CV (%)	SD (pg/mL)	CV (%)	SD (pg/mL)	CV (%)	SD (pg/mL)	CV (%)	SD (pg/mL)	CV (%)
**GFAP Assay**												
L1	240	161.2	6.80	4.22	1.77	1.10	1.78	1.11	2.54	1.57	7.76	4.81
L2	240	4645.0	166.40	3.58	45.04	0.97	45.91	0.99	148.68	3.20	234.60	5.05
**UCH-L1 Assay**												
L1	240	466.2	28.55	6.12	7.27	1.56	6.43	1.38	16.60	3.56	34.75	7.45
L2	240	1597.6	93.98	5.88	39.23	2.46	19.79	1.24	65.97	4.13	124.87	7.82

Linearity

The linearity of GFAP and UCH-L1 assays was established using venous whole blood samples of varying antigen levels that range from below the lower limit of the reportable range to above the upper reportable range for both GFAP and UCH-L1. The study was based on CLSI guidance EP06-Ed2[[11]]. The linearity for both GFAP and UCH-L1 was demonstrated over the reportable range for each assay in the i-STAT TBI cartridge using the i-STAT Alinity instrument. The regression equation for the linear range of the GFAP assay is y=1.01x-3.49. Deviations from linearity were less than or equal to 15%. The regression equation for the linear range of the UCH-L1 assay is y=0.98x+2.66. Deviations from linearity were less than or equal to 10%.

Linearity Across Reportable Range

Assay	Slope	Intercept	r^2	Range (pg/mL)
GFAP	1.01	-3.49	0.9990	24.3 – 11303.9
UCH-L1	0.98	2.66	0.9977	86.4 – 3281.5

Limit of Quantitation

The limit of quantitation (LoQ) is defined as the lowest amount of a measurand in a sample that can be measured with imprecision %CV ≤ 20%. A study to determine LoQ was performed based on CLSI guidance EP17-A2[[12]], adapted to test whole blood samples. The testing was conducted using three (3) cartridge lots that were used to test twelve (12) low analyte levels of GFAP and UCH-L1 biomarkers in venous whole blood samples. The estimated LoQ for the i-STAT TBI test from this study was 47 pg/mL for the GFAP assay and 32 pg/mL for the UCH-L1 assay. Based on the concentration range in the linearity study, the claimed LoQ of the UCH-L1 assay is 87 pg/mL.

High Dose Hook Effect

The GFAP and UCH-L1 assays in the *i-STAT TBI* cartridge on the *i-STAT Alinity* System were evaluated for high dose hook effect. The testing was conducted using venous whole blood samples spiked to a high antigen level for each assay. Each sample was tested to verify that the measured signal is greater than that of a nominal GFAP target of 10,000 pg/mL and a nominal UCH-L1 target of 4000 pg/mL. Hook effect was not observed for the GFAP and UCH-L1 assays using whole blood samples with antigen concentrations exceeding 100,000 pg/mL.

LIMITATIONS OF THE PROCEDURE

- The i-STAT TBI test is not intended to be used as a stand-alone device but as an adjunct to other clinical information to aid in the evaluation of patients who are being considered for standard of care neuroimaging.
- A 'Not Elevated' result is generally associated with the absence of acute intracranial lesions. An appropriate neuroimaging method is required for diagnosis of acute intracranial lesions.
- This device is for use by health care professionals in a point of care or clinical laboratory setting.
- The frequency of suppressed results is affected by atmospheric pressure. Suppressed result rates may increase with higher elevations (decreased barometric pressure) and may become persistent if testing is performed at more than 7500 feet (2286 meters) above sea level. Where unavailability of results is unacceptable, Abbott recommends having an alternate method for excluding traumatic brain injury.
- Samples from patients who have been exposed to animals or who have received therapeutic or diagnostic procedures employing immunoglobulins or reagents derived from immunoglobulins may contain antibodies, e.g., HAMA or other heterophile antibodies, which may interfere with immunoassays and produce erroneous results [13-19]. The generation of potentially interfering antibodies in response to bacterial infections has been reported [15]. While this product contains reagents that minimize the effect of these interferents and QC algorithms designed to detect their effects, the possibility of interference causing erroneous results should be evaluated carefully in cases where there are inconsistencies in the clinical information.
- The instrument must remain on a flat surface with the display facing up during testing. Movement of the instrument during testing may increase the frequency of suppressed results or quality check failures. A level surface includes running the instrument in the base station.
- The test results should be assessed in conjunction with the patient's symptoms, clinical examination, and other findings. If results appear inconsistent with the clinical assessment, the patient sample should be retested using another cartridge.

Factors Affecting Results

Factor	Assay	Effect
Hemolysis	GFAP<br>UCH-L1	Grossly hemolyzed samples can cause a decreased alkaline phosphatase activity, increased assay backgrounds, and/or quality check failures. Increases in UCH-L1 concentration have been observed in hemolyzed samples.
Sample Handling	GFAP<br>UCH-L1	Vortexing and mechanical rotating of the blood sample should be avoided. This type of agitation has been observed to lead to decreases in GFAP concentration and increases in UCH-L1 concentration.
Altitude	GFAP<br>UCH-L1	The i-STAT TBI test has not been evaluated at altitudes >7,500 feet. No impact on performance was found up to 7,500 feet of altitude.

Interference Testing

Interference studies were based on CLSI guideline EP07 3rd edition [20]. The substances listed were evaluated in venous whole blood for relevant assays. For those identified as an interferant the interference is described in the table below. Substances identified below as having no interference had no significant effect (less than 10%) on either the GFAP or UCH-L1 assays.

Interfering Substance Testing

Substance	Test Concentration µmol/L	Test Concentration mg/dL	Assay	Interference (Yes/No)	Comment
Acetaminophen [a]	1324	20	GFAP	No	
			UCH-L1	No	
Acetylsalicylic acid [a]	3620	65.22	GFAP	No	
			UCH-L1	No	
Albumin	150 g/L	15 g/dL	GFAP	No	
			UCH-L1	Yes	Decreased results at >12.1 g/dL. The highest concentration in the reference interval reported by CLSI EP37 is 5.2 g/dL.
Amphetamine	2.44	0.033	GFAP	Yes	Decreased results at >1.83 µmol/L. The highest drug concentration under therapeutic treatment reported by CLSI EP37 is 0.815 µmol/L.
			UCH-L1	No	
Ascorbic acid	298	5.90	GFAP	No	
			UCH-L1	No	
Benzoylecgonine [a]	8.64	2.5 µg/mL	GFAP	No	
			UCH-L1	No	
Bilirubin	684	40	GFAP	No	
			UCH-L1	No	
Bilirubin (conjugated)	475	40	GFAP	No	
			UCH-L1	No	
Caffeine	556	10.8	GFAP	No	
			UCH-L1	No	
Chloramphenicol	241	7.79	GFAP	No	
			UCH-L1	No	
Clopidogrel [a]	21.4	0.90	GFAP	No	
			UCH-L1	No	
Cocaine [a]	3.46 µg/mL	0.346	GFAP	No	
			UCH-L1	Yes	Decreased results at >2.595 µg/mL. The mean maximum plasma concentration (Cmax) per literature is 0.115 µg/mL [[21]].
Diazepam	105	2.99	GFAP	No	
			UCH-L1	No	
Diclofenac	81	2.58	GFAP	No	
			UCH-L1	No	
Dopamine	4.06	0.077	GFAP	No	
			UCH-L1	No	
EDDP† [a]	0.33	125 ng/mL	GFAP	No	
			UCH-L1	No	
Erythromycin	188	13.80	GFAP	No	
			UCH-L1	No	
Ethanol	130 mmol/L	599	GFAP	No	

Substance	Test Concentration µmol/L	Test Concentration mg/dL	Assay	Interference (Yes/No)	Comment
			UCH-L1	No	
Hemoglobin	10 g/L	1000	GFAP	No	
			UCH-L1	No	
Human anti-mouse antibodies (HAMA) [a]	>80x [b]	N/A	GFAP	No	
			UCH-L1	No	
Ibuprofen [a]	2425	50.0	GFAP	No	
			UCH-L1	No	
Intralipid (Intralipid 20%)	N/A	7075	GFAP	No	
			UCH-L1	No	
Methadone	10.3	0.319	GFAP	Yes	Decreased results at >7.725 µmol/L. The highest drug concentration under therapeutic treatment reported by CLSI EP37 is 3.43 µmol/L.
			UCH-L1	No	
d-Methamphetamine [a]	1.86	278.3 ng/mL	GFAP	Yes	Decreased results at >208.8 ng/mL. The mean maximum plasma concentration (Cmax) per literature is 92.8 ng/mL.[[22]]
			UCH-L1	No	
Methaqualone [a]	32.36	8.1 µg/mL	GFAP	No	
			UCH-L1	No	
Metoprolol [a]	18.7	1.28	GFAP	No	
			UCH-L1	Yes	Decreased results at >14.025 µmol/L. The highest drug concentration under therapeutic treatment reported by CLSI EP37 is 1.875 µmol/L.
Morphine	27.3	0.78	GFAP	No	
			UCH-L1	No	
Nicardipine hydrochloride	0.97	0.05	GFAP	No	
			UCH-L1	No	
Nicotine	5.97	0.097	GFAP	No	
			UCH-L1	No	
Oxazepam	15.1	0.43	GFAP	No	
			UCH-L1	No	
Phencyclidine [a]	0.0357	8.7 ng/mL	GFAP	No	
			UCH-L1	No	
Phenytoin	238	6.0	GFAP	No	
			UCH-L1	No	
Propoxyphene [a]	9.46	0.32	GFAP	No	
			UCH-L1	Yes	Decreased results at >7.095 µmol/L. The highest drug concentration under therapeutic treatment reported by CLSI EP37 is 3.15 µmol/L.
Rheumatoid Factor (RF) [a]	1000 IU/mL	N/A	GFAP	No	

Substance	Test Concentration		Assay	Interference	Comment
	µmol/L	mg/dL		(Yes/No)	
			UCH-L1	Yes	Decreased results at >875 IU/mL
Secobarbital	66.8	1.59	GFAP	No	
			UCH-L1	No	
Triglycerides [a]	33.88 mmol/L	3000	GFAP	No	
			UCH-L1	No	
Warfarin	243	7.5	GFAP	No	
			UCH-L1	No	

†2-Ethylidene-1,5-dimethyl-3,3-diphenylpyrrolidine

[a] The test concentration used for this substance is not from CLSI guideline EP37 1st edition [23]

[b] The 'x' factor listed indicates the number of times more activity than a known negative sample for its ability to crosslink antibodies in a mouse system assay.

[c] One out of the five samples enriched for presence of RF exhibited an interference effect. See note regarding HAMA or other heterophile antibodies in Limitations of the Procedure section above.

This is representative data and results may vary from study to study due to matrix effects. Viscosity, surface tension, turbidity, ionic strength, and pH are common causes of matrix effects. It is possible that interfering substances other than those tested may be encountered. The degree of interference at concentrations other than those listed has not been tested.

Analytical Specificity

The i-STAT TBI cartridge is specific to the measurement of glial fibrillary acid protein (GFAP) and ubiquitin carboxyl-terminal hydrolase L1 (UCH-L1). The following proteins in the table below with significant homology to GFAP or UCH-L1 were tested at highest known physiological levels and none were found to have significant impact on the measured GFAP or UCH-L1 levels.

Cross-Reactivity Testing

Substance	Test Concentration pg/mL	Assay	Cross-reactivity (Yes/No)
Keratin type II	10 000	GFAP	No
Internexin	77 000	GFAP	No
Neurofilament medium	8 600	GFAP	No
Neurofilament heavy	77 000	GFAP	No
Neurofilament light	68	GFAP	No
Peripherin	5 000	GFAP	No
Desmin	127 000	GFAP	No
Vimentin	354 000	GFAP	No
Ubiquitin Carboxyl-Terminal Hydrolase L3 (UCH-L3)	354 000	UCH-L1	No

Hematocrit Sensitivity

The assays on the i-STAT TBI cartridge have been characterized in venous whole blood samples with hematocrit levels up to 60% PCV. Imprecision (CV) and bias exceeding 10% have been observed for samples with hematocrit levels above 56% PCV.

KEY TO SYMBOLS

Symbol	Definition/Use
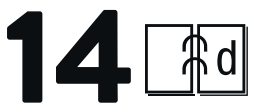	14 days room temperature storage at 18–30 °C.
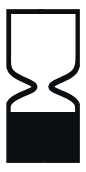	Use by or expiration date.<br>The expiration date, expressed as YYYY-MM-DD, indicates the last day the product may be used.
LOT	Manufacturer's lot number or batch code.<br>The lot number or batch code appears adjacent to this symbol.
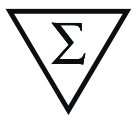	Sufficient for <n> tests.
EU REP	Authorized representative in the European Community
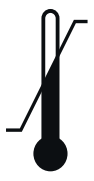	Temperature limitations.<br>The upper and lower limits for storage are adjacent to upper and lower arms.
REF	Catalog number, list number, or reference.
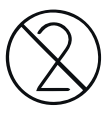	Do not reuse.
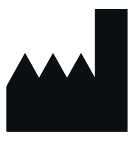	Manufacturer.
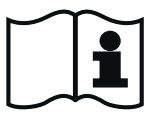	Consult instructions for use or see System Manual for instructions.
IVD	*in vitro* diagnostic medical device.
CE 0344	A mark that indicates conformity to the legal requirements of the appropriate European Union (EU) Directive(s) and Regulation(s) with respect to safety, health, environment and consumer protection.
Rx ONLY	For prescription use only.
i-STAT Alinity ONLY	For use with i-STAT Alinity System only.
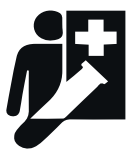	Device for near-patient testing
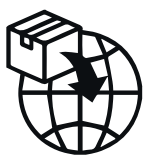	Importer in the European Community
UK CA	UK Conformity Assessed mark

Additional Information: to obtain additional product information and technical support, refer to the Abbott company website at www.globalpointofcare.abbott

Product issues and adverse events should be reported to Abbott through your Abbott Point of Care support service. For a patient/user/third party in the European Union and in countries with identical regulatory regime (Regulation 2017/746/EU on In vitro Diagnostic Medical Devices); if, during the use of this device or as a result of its use, a serious incident has occurred, please report it to Abbott and its authorised representative and to your national authority.

REFERENCES

1. Levine, Z. (2010) Mild traumatic brain injury: part 1: determining the need to scan. *Can Fam Physician.* **56**, 346-349
2. Smith-Bindman, R., Lipson, J., Marcus, R., Kim, K. P., Mahesh, M., Gould, R. *et al.* (2009) Radiation dose associated with common computed tomography examinations and the associated lifetime attributable risk of cancer. *Arch Intern Med.* **169**, 2078-2086
3. Bazarian, J. J., Biberthaler, P., Welch, R. D., Lewis, L. M., Barzo, P., Bogner-Flatz, V. *et al.* (2018) Serum GFAP and UCH-L1 for prediction of absence of intracranial injuries on head CT (ALERT-TBI): a multicentre observational study. *Lancet Neurol.* **17**, 782-789
4. Jones, A., and Jarvis, P. (2017) Review of the potential use of blood neuro-biomarkers in the diagnosis of mild traumatic brain injury. *Clin Exp Emerg Med.* **4**, 121-127
5. Metting, Z., Wilczak, N., Rodiger, L. A., Schaaf, J. M., and van der Naalt, J. (2012) GFAP and S100B in the acute phase of mild traumatic brain injury. *Neurology.* **78**, 1428-1433
6. Papa, L., Lewis, L. M., Falk, J. L., Zhang, Z., Silvestri, S., Giordano, P. *et al.* (2012) Elevated levels of serum glial fibrillary acidic protein breakdown products in mild and moderate traumatic brain injury are associated with intracranial lesions and neurosurgical intervention. *Ann Emerg Med.* **59**, 471-483
7. Tongaonkar, P., Chen, L., Lambertson, D., Ko, B., and Madura, K. (2000) Evidence for an interaction between ubiquitin-conjugating enzymes and the 26S proteasome. *Mol Cell Biol.* **20**, 4691-4698
8. Papa, L., Lewis, L. M., Silvestri, S., Falk, J. L., Giordano, P., Brophy, G. M. *et al.* (2012) Serum levels of ubiquitin C-terminal hydrolase distinguish mild traumatic brain injury from trauma controls and are elevated in mild and moderate traumatic brain injury patients with intracranial lesions and neurosurgical intervention. *J Trauma Acute Care Surg.* **72**, 1335-1344
9. CLSI. *Defining, Establishing, and Verifying Reference Intervals in the Clinical Laboratory.* 3rd ed. CLSI document EP28-A3c. Wayne, PA: Clinical and Laboratory Standards Institute; 2008
10. CLSI. *Evaluation of Precision of Quantitative Measurement Procedures.* 3rd ed. CLSI document EP05-A3. Wayne, PA: Clinical and Laboratory Standards Institute; 2014
11. CLSI. *Evaluation of the Linearity of Quantitative Measurement Procedures*. 2nd ed. CLSI guideline EP06, Wayne, PA: Clinical and Laboratory Standards Institute; 2020.
12. CLSI. *Evaluation of Detection Capability for Clinical Laboratory Measurement Procedures.* 2nd ed. CLSI document EP17-A2. Wayne, PA: Clinical and Laboratory Standards Institute; 2012
13. Bjerner, J., Nustad, K., Norum, L. F., Olsen, K. H., and Bormer, O. P. (2002) Immunometric assay interference: incidence and prevention. *Clin Chem.* **48**, 613-621
14. Boscato, L. M., and Stuart, M. C. (1988) Heterophilic antibodies: a problem for all immunoassays. *Clin Chem.* **34**, 27-33
15. CLSI. *Immunoassay Interference by Endogenous Antibodies; Approved Guidelines* . CLSI document I/LA30-A. Wayne, PA: Clinical and Laboratory Standards Institute; 2008
16. Kricka, L. J. (2000) Interferences in immunoassay--still a threat. *Clin Chem.* **46**, 1037-1038
17. Nahm, M. H., and Hoffmann, J. W. (1990) Heteroantibody: phantom of the immunoassay. *Clin Chem.* **36**, 829
18. Primus, F. J., Kelley, E. A., Hansen, H. J., and Goldenberg, D. M. (1988) "Sandwich"-type immunoassay of carcinoembryonic antigen in patients receiving murine monoclonal antibodies for diagnosis and therapy. *Clin Chem.* **34**, 261-264
19. Schroff, R. W., Foon, K. A., Beatty, S. M., Oldham, R. K., and Morgan, A. C., Jr. (1985) Human anti-murine immunoglobulin responses in patients receiving monoclonal antibody therapy. *Cancer Res.* **45**, 879-885
20. CLSI. *Interference Testing in Clinical Chemistry*. 3rd ed. CLSI document EP07. Wayne, PA: Clinical and Laboratory Standards Institute; 2018
21. Scheidweiler, K. B., Spargo, E. A., Kelly, T. L., Cone, E. J., Barnes, A. J., and Huestis, M. A. (2010) Pharmacokinetics of cocaine and metabolites in human oral fluid and correlation with plasma concentrations after controlled administration. *Ther Drug Monit.* **32**, 628-637

22. Karch, S. (2008) Dissociative Anesthetics. In: *Karch's Pathology of Drug Abuse*, 4th ed. Boca Raton, FL: CRC Press
23. CLSI. *Supplemental Tables for Interference Testing in Clinical Chemistry*. 1st ed. CLSI supplement EP37. Wayne, PA: Clinical and Laboratory Standards Institute; 2018

i-STAT and Alinity are trademarks of Abbott.
All trademarks referenced are trademarks of either Abbott or their respective owners.

Abbott Point of Care Inc.
400 College Road East
Princeton, NJ 08540 USA

UKCA

UK Responsible Person
Emergo Consulting (UK) Limited
c/o Cr 360 – UL International
Compass House, Vision Park Histon
Cambridge CB24 9BZ
United Kingdom

CE 0344

IVD

Rx ONLY

Technical Support: please contact your local service provider for service information.

For customers in the European Union: A summary of safety and performance (SSP) for this device is available at https://ec.europa.eu/tools/eudamed/ after the launch of the European Database on Medical Devices. Search for the device using the UDI-DI provided on the outer packaging of the device. A copy of the SSP can also be requested from the European Authorized Representative or the manufacturer.

©2025 Abbott Point of Care Inc. All rights reserved. Printed in Germany.
